# Comprehensive mechanical reinforcement of hydrogels via network refinement

**DOI:** 10.1016/j.isci.2026.116191

**Published:** 2026-06-01

**Authors:** Han Li, Zidi Zhou, Yuan Gao, Jincheng Lei, Zishun Liu

**Affiliations:** 1 International Center for Applied Mechanics, State Key Laboratory for Strength and Vibration of Mechanical Structures, Xi’an Jiaotong University, Xi’an 710049, China; 2 City University of Hong Kong (Dongguan), Dongguan 523808, China; 3 City University of Hong Kong, Hong Kong 999077, China

**Keywords:** Materials science, Mechanical property, Polymers

## Abstract

Traditional single-network hydrogels are limited by trade-offs among key mechanical properties, such as elastic modulus and toughness, and are further compromised by structural defects introduced during synthesis. These constraints significantly hinder their performance in demanding applications. Here, we propose a network-refinement strategy using repeated crosslinking to achieve comprehensive mechanical reinforcement. Through network refinement, structural defects within polymer networks are progressively filled, and both the network homogeneity and effective chain density are improved. The reinforced hydrogels exhibit up to a 6-fold increase in elastic modulus, a 10-fold enhancement in fracture toughness, a 20-fold increase in tensile strength, and a 42-fold improvement in work of fracture, while maintaining high stretchability and negligible hysteresis under moderate deformation. This universal strategy provides an effective route to comprehensively enhance the mechanical properties of hydrogel-like materials, paving the way for robust soft materials in applications such as cardiac healing patches, load-bearing biomedical implants, and wearable electronics.

## Highlights

• Repeated crosslinking fills defects and homogenizes hydrogel networks
• Entangled slip-links redistribute stress and delay crack propagation
• RC hydrogels gain 6*x* modulus, 10*x* toughness, 20*x* strength and 42*x* work of fracture
• Chemistry-independent refinement enables hydrogels for implants and wearables

## Graphical abstract

**Figure:**
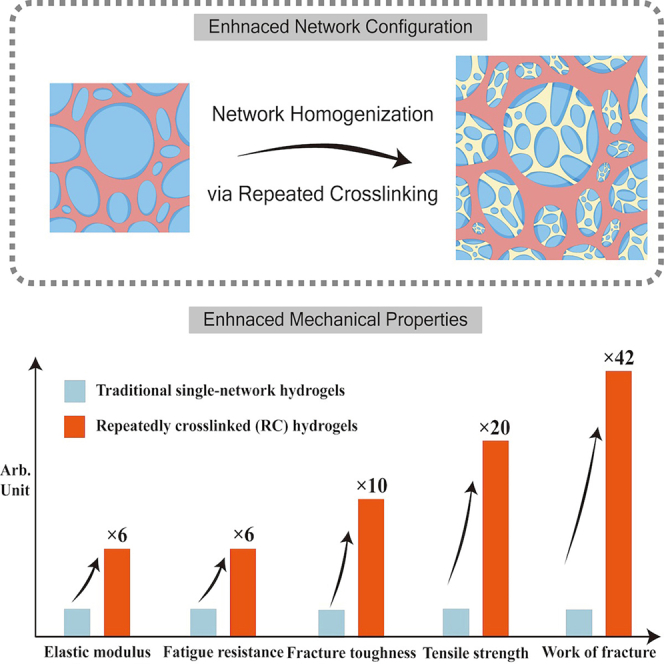

## Introduction

Just as bones provide structural support to the human body, polymer networks serve as the structural backbone of soft materials like hydrogels and elastomers, ensuring material integrity while enabling significant deformation.^1^^,^^2^ However, the inherent limitations of polymer networks, coupled with unavoidable imperfections introduced during radical polymerization, undermine their mechanical performance.^3^^,^^4^^,^^5^^,^^6^ In an ideal single-network (SN) polymer, where chains are uniformly distributed, of equal length, stretched identically, and rupture simultaneously, theoretical predictions indicate that both the elastic modulus *E* and fatigue threshold *G*_th_ depend on the chain length *n*, but in opposite ways: *E* scales inversely with *n* (*E* ∼ 1/*n*), while *G*_th_ scales with the square root of *n* ($G_{\text{th}}∼\sqrt{n}$).^7^^,^^8^^,^^9^ This inverse relationship creates a fundamental design challenge: an SN polymer cannot simultaneously achieve a high elastic modulus and a high fatigue threshold. This trade-off is commonly referred to as modulus-threshold conflict. Similar trade-offs are observed among other mechanical properties of soft materials, e.g., strength and toughness,^10^ modulus and toughness,^11^ or stretchability and reversibility.^12^ These inherent compromises impose significant constraints on material design and performance optimization. Furthermore, real polymer networks are widely acknowledged to be structurally imperfect,^13^^,^^14^^,^^15^ and are often characterized by unequal chain lengths, dangling ends, closed loops, and voids. For example, chain-length heterogeneity is believed to improve fracture toughness by facilitating energy dissipation through distributed chain scission.^14^^,^^16^ However, this improvement typically comes at the expense of strength and stretchability^14^^,^^17^^,^^18^^,^^19^ while offering little contribution to the fatigue threshold.^14^^,^^16^ Consequently, the imbalance among competing mechanical properties becomes even more pronounced.

To address the limitations in the mechanical properties of polymers, especially hydrogels, researchers have explored two main strategies: enhancing the uniformity of a single polymer network^20^^,^^21^ or introducing complementary mechanisms that independently enhance different mechanical properties.^22^^,^^23^^,^^24^^,^^25^ In the first approach, tetra-arm poly (ethylene glycol) (tetra-PEG) hydrogels^20^^,^^21^ have been developed due to their ability to synthesize polymer chains with equal lengths, uniform composition, and minimal defects. While this structural regularity enables precise control, the resulting mechanical performance remains constrained, typically achieving an elastic modulus around 10 kPa and a fatigue threshold near 10 J/m^2^,^26^ which is still insufficient for many practical applications. By contrast, the second strategy provides more robust mechanical performance by integrating mechanisms that balance competing properties more effectively. A classic example is the double-network strategy,^27^ which combines a stiff, brittle, short-chain network with a soft, stretchable, long-chain network. The short chains contribute to a high elastic modulus of ∼1000 kPa, while the long chains enhance the fatigue threshold to ∼100 J/m^2^.^28^ However, the sacrificial breaking of the short chains results in significant hysteresis, underscoring that the trade-off persists despite the improved fatigue threshold. A similar strategy has also been applied to elastomers to construct double and triple networks via sequential polymerization.^29^^,^^30^ These elastomers contain a minority of isotopically prestretched chains in the first network, which are designed to rupture preferentially at the crack tip. This sacrificial fracture serves as the core energy dissipation mechanism, enabling a simultaneous increase in both modulus and fracture energy.^31^ Sun et al. embedded rhodamine-based mechanophores in multi-network elastomers and, via cycle-to-cycle fluorescence comparison, mapped first-cycle stress and quantified accumulated damage; combined with modeling and finite-element simulations, this offers a concise and highly practical route to visualize stress/damage under complex deformations.^32^ The recently developed entangled networks^12^^,^^33^^,^^34^^,^^35^ is another example of the second strategy. These materials feature a much higher density of physical entanglements compared to chemical crosslinks. Under deformation, the elastic modulus is governed by the segment length between entanglements, while the fatigue threshold remains associated with the chain end-to-end length. This design achieves an elastic modulus of ∼100 kPa and a fatigue threshold of ∼200 J/m^2^.^12^ Yet, these structures often suffer from limited stretchability. While each of these strategies has pushed the boundaries of polymeric materials, none has yet succeeded in achieving comprehensive enhancement across multiple mechanical properties—including elastic modulus, toughness, elasticity, fatigue resistance, and more—within a single material system.

**Figure 1:**
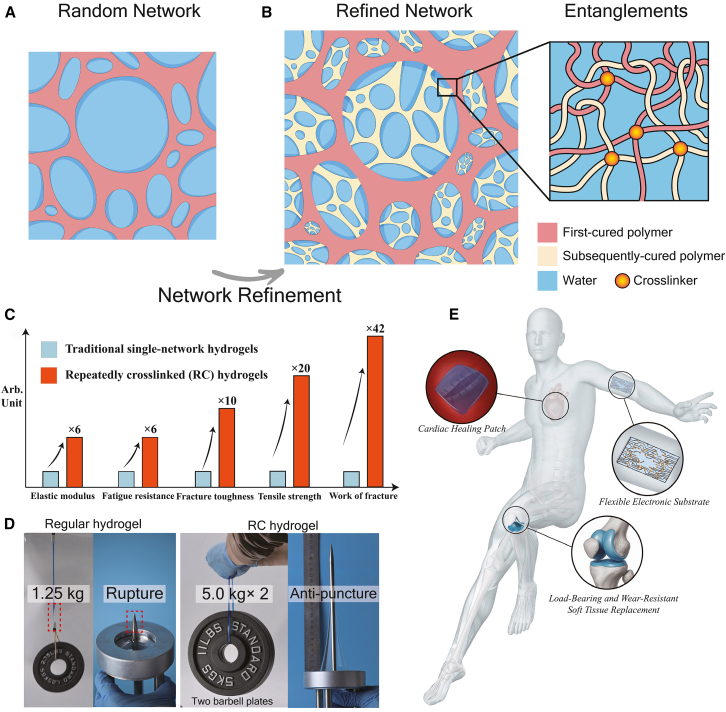
Network refinement strategy for mechanical reinforcement of hydrogels (A) Schematic of network structure in a conventional SN hydrogel, showing an inhomogeneous structure with defects like large voids. (B) Schematic of repeated crosslinking strategy, yielding the resulting RC hydrogel with multiple interpenetrating networks and improved homogeneity. The inset depicts the physical entanglement between chemically identical networks. (C) Quantitative enhancement of the mechanical properties of RC hydrogels compared with SN hydrogels. Elastic modulus and fatigue resistance increase nearly 6-fold, fracture toughness nearly 10-fold, tensile strength 20-fold, and work of fracture 42-fold. (D) Demonstration of tensile and puncture resistance. RC hydrogels can lift 10-kg barbells and resisted severe puncture without failure, whereas SN hydrogels failed. (E) Potential applications of RC hydrogels in cardiac healing patches, load-bearing and wear-resistant soft tissue replacements, and flexible electronic substrates.

In this work, we introduce a simple yet universal and chemistry-independent strategy called repeated crosslinking, which is designed to simultaneously enhance elastic modulus, strength, work of fracture, toughness, fatigue life, wear resistance, and puncture resistance, while maintaining negligible hysteresis in hydrogels. Using polyacrylamide (PAAm) hydrogels as a model system, we demonstrate how this strategy yields a repeatedly crosslinked (RC) hydrogel and enables comprehensive mechanical reinforcement. As illustrated in Figure 1A, conventional SN hydrogels synthesized via radical polymerization typically form inhomogeneous networks containing numerous structural defects. These defects act as stress concentrators during deformation, locally amplifying mechanical loads and causing premature failure. Our strategy addresses these challenges by repeatedly swelling the hydrogel with the precursor solution and initiating polymerization within the preexisting network(s). Each cycle introduces new polymer chains that fill voids and defects, progressively refining the network architecture to achieve a more homogeneous and robust structure. This refinement results in a hydrogel composed of multiple interpenetrating networks, as shown in Figure 1B. These networks are physically entangled yet chemically continuous, forming a dense and coherent matrix. The magnified schematic on the right of Figure 1B highlights the smooth integration between successive networks, underscoring the structural integrity achieved through this approach. Notably, this structural evolution results in substantial enhancements in mechanical performance. As shown in Figure 1C, the RC hydrogel exhibits remarkable improvements across all measured properties. Specifically, the elastic modulus increases to 25 kPa, representing a nearly 6-fold improvement over the original lightly crosslinked SN hydrogel; fracture toughness increases almost 10-fold, from 272 J/m^2^ to 2800 J/m^2^; tensile strength reaches 700 kPa, a 20-fold enhancement; the work of fracture rises from 118 kJ/m^2^ to 4991 kJ/m^2^, indicating a nearly 42-fold increase (Figure S1). The enhancement in tensile strength and puncture resistance is further demonstrated in Figure 1D and Video S1: unlike the original SN hydrogel, the RC hydrogel is capable of lifting 10-kg barbells and withstand severe puncture without failure. The comprehensively enhanced mechanical properties achieved by this strategy are essential for broadening the applications of hydrogels. In particular, as shown in Figure 1E, the combination of high strength, fracture toughness, fatigue resistance, wear resistance, and large-deformation capacity greatly expands their prospects in areas such as human health monitoring, load-bearing biomedical implants, and wearable electronics.

## Results

### Uniaxial tensile behavior and microstructure of RC hydrogels

**Figure 2:**
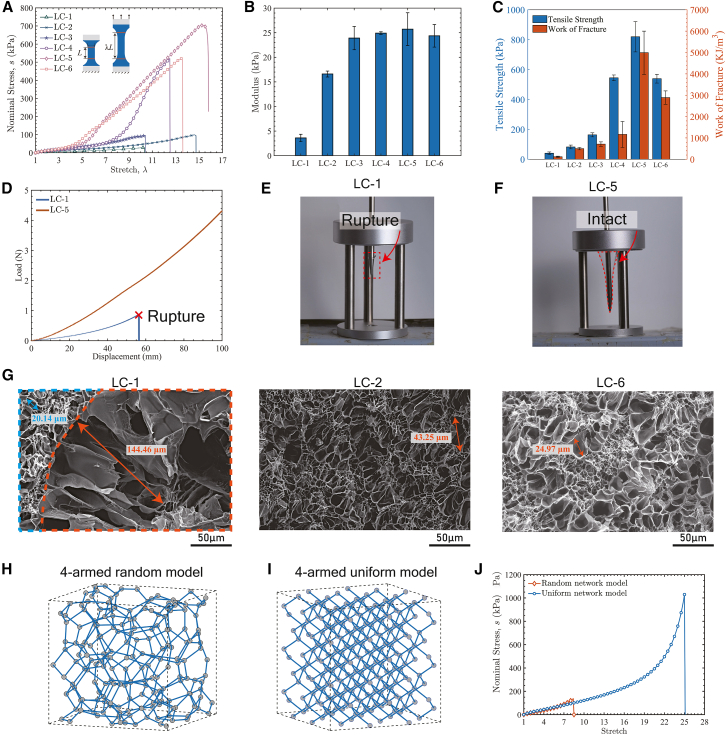
Mechanical behavior and microstructure of RC hydrogels (A) Nominal stress-stretch curves of LC hydrogels under uniaxial tension. (B) Elastic modulus of LC hydrogels. Data are presented as mean ± SD (*n* = 5). (C) Tensile strength and work of fracture of LC hydrogels. Data are presented as mean ± SD (*n* = 5). (D) Nominal stress-displacement curves of LC-1 and LC-5 under puncture. LC-1 ruptured at a displacement of 56.15 mm, whereas LC-5 remained intact even at 100.0 mm (the maximum displacement allowed by the test apparatus). (E–F) Photographs of LC-1 and LC-5 at displacements of 56.15 mm and 100.0 mm, respectively, during puncture. (G) Microstructure of RC hydrogels observed via scanning electron microscopy (SEM). LC-1 exhibited a bimodal pore distribution, with large pores (144.46 μm, orange dashed outline) surrounded by significantly smaller pores (blue dashed outline), indicating pronounced structural heterogeneity. The second crosslinking cycle reduced the maximum pore size in LC-2 to 43.25 μm, reflecting network improvement. LC-6 exhibited a more uniform porous structure with a maximum pore size of only 24.97 μm, demonstrating that repeated crosslinking progressively uniformizes the network. Scale bars in (G): 50 μm. (H–J) Random and uniform mesoscopic network models and their corresponding nominal stress-stretch curves under uniaxial tension.

A series of RC hydrogels was synthesized with varying initial crosslinking densities and numbers of successive crosslinking cycles. The fabrication process of RC hydrogels is illustrated in Figure S2 and summarized in Table S1 (details are provided in the Experimental Section). We use LC-x, MC-x, and HC-x to refer to low-, medium-, and high-crosslinked hydrogels, respectively, with x (where x = 1, 2, 3, …) successive crosslinked networks. Dumbbell-shaped hydrogel specimens with different crosslinking densities (LC/MC/HC) were subjected to uniaxial tensile tests under quasi-static conditions to evaluate their stress–stretch behavior. Representative curves for the LC series are shown in Figure 2A, while corresponding data for the MC and HC series are presented in Figure S3. From these Figures, the initial elastic modulus, tensile strength, and work of fracture were extracted, as shown in Figures 2B and 2C, S4, S5. Furthermore, the puncture resistance is increased, as shown in Figures 2D–2F. Across all series, repeated crosslinking consistently enhances the initial elastic modulus, tensile strength, and work of fracture relative to the original SN hydrogel.

According to polymer network theory, the initial elastic modulus *E*_initial_ is directly proportional to the polymer chain density^36^^,^^37^ and inversely proportional to the density of defects within the network.^38^^,^^39^ Conventional SN hydrogels, synthesized via single-step polymerization, exhibit low polymer chain density and numerous structural defects such as voids and dangling chains, which severely decrease the elastic modulus.^14^^,^^40^^,^^41^ Repeated crosslinking introduces interpenetrating networks that, on one hand, increase the effective polymer chain density, and on the other hand, reduce defects by filling voids and connecting dangling chains through entangled polymer chains, thereby increasing the elastic modulus of hydrogels. However, after three repeated crosslinking cycles (LC-4), the hydrogel network reaches an optimal structure in which most structural voids are eliminated. Subsequent crosslinking cycles introduce only a limited number of new polymer chains. These additional chains are relatively relaxed and contribute minimally to load bearing, leading to a plateau in the elastic modulus beyond LC-4.

Scanning electron microscopy (SEM) observations of hydrogels with different crosslinking cycles further support the previous mechanism of microstructural evolution during repeated crosslinking. As shown in Figure 2G, SEM images of the LC-1 reveal a distinctly bimodal pore size distribution, characterized by large pores (outlined in the orange dashed box) with a maximum diameter of approximately 144.46 μm, and smaller pores (outlined in the blue dashed box) measuring less than one-third that size, around 20.14 μm. After one additional crosslinking cycle, the maximum pore size of LC-2 decreases significantly to 43.25 μm. With further crosslinking, the network structure of LC-6 becomes even more refined, with the largest pores reduced to 24.97 μm, and the pore size distribution is more uniform. The low-magnification SEM images of these hydrogel samples are also presented in Figure S15.

In LC series hydrogels, tensile strength progressively increases over the first five crosslinking cycles, peaking at LC-5, as shown in Figure 2C. A similar increasing trend in tensile strength is also observed in the MC and HC series hydrogels (Figure S5). The tensile strength of a hydrogel is closely related to the density of effective load-bearing polymer chains at the fracture stretch. As mentioned earlier, the additional crosslinked network entangles with the original network, thereby increasing the effective polymer chain density. Beyond a critical number of crosslinking cycles (LC-5), the mobility of polymer chains becomes increasingly restricted due to excessive entanglement between networks. This restriction results in the premature fracture of a large number of polymer chains at small stretches, which reduces the density of effective polymer chains and subsequently lowers the tensile strength of hydrogels. The stretchability of the LC series hydrogels exhibits a similar non-monotonic trend, first increasing before decreasing at higher cycle numbers. In contrast, for MC and HC hydrogels, the stretchability continuously declines with each additional network introduced (Figure S6). This is because the original polymer chains in MC and HC hydrogels are relatively short, and the newly introduced cured polymer networks readily restrict the mobility of polymer chains, thereby decreasing stretchability. By comparison, LC hydrogels initially possess relatively long polymer chains, allowing the newly formed polymer networks to occupy free voids and reduce structural heterogeneity, which enhances stretchability during the initial crosslinking cycles. Only after several crosslinking cycles does the restrictive effect of additional polymer networks on the preexisting chains become evident in LC hydrogels. As a combined outcome of strength and stretchability, the work of fracture of hydrogels finally exhibits an initial increase followed by a subsequent decrease with increasing crosslinking cycles. The enhancement of the work of fracture in RC hydrogels with an appropriate number of crosslinking cycles is further demonstrated by puncture tests. Figures 2D–2F shows the load-displacement curves of LC-1 and LC-5 during the puncture test, together with photographs of the specimens on the verge of rupture. LC-1 failed at a spike displacement of 56.2 mm, whereas LC-5 remained intact at the maximum displacement of 100.0 mm, which was limited by the apparatus capacity.

To provide additional insights into how network uniformity enhances hydrogel stretchability, we constructed three-dimensional polymer networks with either uniform or random configurations within a periodic cubic domain (Figures 2H and 2I), using a mesoscopic network model (see Supporting Methods S1 for modeling details and simulation procedures). Simulation results demonstrate that the uniform network exhibits higher stretchability and tensile strength than the random network under uniaxial tension (Figure 2J). The random network structure generates local stress concentrations during stretching, leading to premature failure of the overall structure. In contrast, the homogeneous network structure reduces weak points such as voids and defects, enabling polymer chains to distribute external loads more effectively without localized stress concentrations, thereby significantly enhancing both stretchability and tensile strength.

Based on the preceding analysis, optimizing the number of crosslinking cycles is critical for maximizing the mechanical performance of hydrogels. In the LC-1 hydrogel (Figure S7), polymer chains formed via free-radical polymerization are randomly distributed, resulting in nonuniform stress transfer under external stretching. Some chains remain inactive in load-bearing, while others rupture prematurely at localized stress concentrations (highlighted in red). With an appropriate number of repeated crosslinking cycles, as demonstrated by the LC-5 (Figure S7), pre-existing defects, such as large voids and dangling chains, are mitigated due to the continuous filling of newly added networks, which facilitates more uniform stress distribution. Additionally, the slippage mechanism of entangled polymer chains facilitates stress redistribution from overstressed regions (red chains in LC-5) to adjacent chains, further reducing local stress concentrations. Furthermore, the effective polymer chain density is significantly improved. Consequently, LC-5 demonstrates substantial improvements in modulus, tensile strength, stretchability, and work of fracture. However, excessive crosslinking, exemplified by the LC-6 hydrogel (Figure S7), results in overly entangled polymer networks with restricted chain mobility. Upon stretching, the excessive entanglement of these chains may not contribute positively to the mechanical properties of hydrogels. Instead, it may restrict the mobility of polymer chains and trigger early chain scission, thereby reducing the density of load-bearing chains and ultimately leading to a plateau or even deterioration in mechanical properties.

### Effects of pre-stretch and entanglement on RC hydrogels

**Figure 3:**
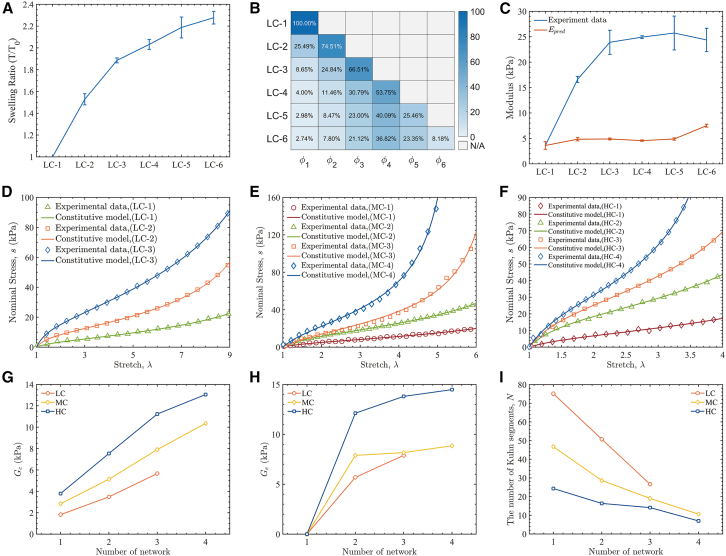
Hyperelastic entanglement model quantifies entanglement-driven reinforcement in RC hydrogels (A) The pre-stretch ratio of the first network (*λ*_0,1_) in different LC series hydrogels. Data are presented as mean ± SD (*n* = 5). (B) The mass fractions of the *i*^*th*^ network (*ϕ*_*i*_) for each LC series hydrogel (e.g., for LC-3, the mass fraction of the first network is 8.65% (*ϕ*_1_), 24.84% (*ϕ*_2_) for the second network, and 66.51% (*ϕ*_3_) for the third network). (C) Comparison of experimentally derived initial elastic modulus (*E*_0_) with the pre-stretch-based model prediction (*E*_pred_). Data are presented as mean ± SD (*n* = 5). (D–F) Stress-stretch (*σ* – *λ*) response obtained from both experiments (symbols) and hyperelastic entanglement model (lines) across varying number of networks. (G–I) Fitted model parameters with the change of the number of networks: crosslink-derived shear modulus (*G*_*c*_), entanglement modulus (*G*_*e*_), and the mean number of Kuhn segments (*N*).

During our repeated crosslinking process, each crosslinking cycle first allows the pre-synthesized polymer network to swell to equilibrium in the precursor solution before curing. This process inevitably imposes a pre-stretching on the existing networks.^42^ For cycle *i*, we measured the swelling stretch *λ*_0,*i*_ (the pre-stretch of the embedded network, Figure 3A) and the mass fractions of the *i*^*th*^ network (*ϕ*_*i*_) for each LC-1 to LC-6 hydrogel (Figure 3B). Following Ducrot and Creton,^31^ considering the pre-stretch effect, the effective modulus can be written as

(Equation 1) $$\begin{array}{c} E_{\text{pred}}=\left(\sum_{i}\phi_{i}\lambda_{0,i}^{2}\right)E_{\text{SN}}\text{,} \end{array}$$

where *E*_SN_ denotes the corresponding modulus of SN network hydrogel. However, comparison to experiment (Figure 3C) shows that *E*_pred_ underpredicts the modulus and fails to capture the monotonic increase with the number of networks. We therefore conclude that swelling-induced pre-stretch is not the primary mechanism governing reinforcement in these RC hydrogels. Instead, we propose that repeated crosslinking results in a high density of physical entanglements—effectively slip-links—between interpenetrating networks. These reversible slip-links increase the effective load-bearing chain density and enable cooperative load sharing across networks under deformation. Unlike physical crosslinks, they can rearrange under load, stabilizing deformation and suppressing stress localization; consequently, both modulus and strength increase. We adopted a two-component hyperelastic model, following the framework of Xiang et al.,^43^ to describe the network elasticity. The model decouples the Helmholtz free-energy density (*W*) into contributions from permanent chemical crosslinks (*W*^net^) and physical entanglements (*W*^tube^):

(Equation 2) $$\begin{array}{c} W=W^{\text{net}}+W^{\text{tube}}\text{.} \end{array}$$

The *W*^net^ term, representing the entropic elasticity of the cross-linked network, is described by the Langevin statistical model^44^:

(Equation 3) $$\begin{array}{c} W^{\text{net}}\left(I_{1},N\right)=G^{c}\left(\sqrt{N}\;\beta \;\sqrt{\frac{I_{1}}{3}}+Nln\;\ln\frac{\beta }{\sinh\;\beta }\right)\text{.} \end{array}$$

The *W*^tube^ term accounts for the free energy from topological constraints imposed by chain entanglements, which is represented here by a neo-Hookean form:

(Equation 4) $$\begin{array}{c} W^{\text{tube}}=3G^{e}\sqrt{\frac{I_{2}}{3}}\text{.} \end{array}$$

In these equations, *G*_*c*_ and *G*_*e*_ are the shear moduli for the cross-linked and entanglement networks, respectively. *I*_*c*_ = tr(*C*) is the first invariant of the right Cauchy-Green tensor (*C* = *F*^T^*F*), *N* is the mean number of Kuhn segments per strand, and $\beta =L^{-1}\left(\sqrt{I_{1}/3N}\right)$ is the inverse Langevin function. The material is assumed to be incompressible (*J* = *det*(F) = 1). The principal Cauchy stresses (*σ*_*i*_) are derived from the total free energy:

(Equation 5) $$\begin{array}{c} \sigma_{i}=\lambda_{i}\frac{\partial W}{\partial \lambda_{i}}-p\text{.} \end{array}$$

Here, *p* is the Lagrange multiplier enforcing incompressibility. From this constitutive relation, the uniaxial nominal stress-stretch response was derived (converting from Cauchy to first Piola-Kirchhoff stress) for direct comparison with experimental data. This model was fit to the first-cycle nominal stress-stretch curves for the LC, MC, and HC series via a least-squares method, using only data from stretches without discernible hysteresis or damage (Figures 3D–3F). The model successfully captures the linear response at small strains and the characteristic strain-hardening at large stretches. The fitted parameters *G*_*c*_, *G*_*e*_, and *N* are shown in Figures 3G–3I. For single-network hydrogels (LC-1/MC-1/HC-1) *G*_*e*_ ≈ 0, indicating negligible entanglement-derived elasticity. With each added network, both *G*_*c*_ and *G*_*e*_ increase. Notably, with additional network(s), the value of *G*_*e*_ increases rapidly, reflecting a rapid increase in the number of polymer chains entangled between networks. Similarly, the entangled polymer chains restrict polymer chain movement, leading to a decrease in the average number of Kuhn segments (*N*) in the network. At higher cycle numbers, *G*_*e*_ approaches a plateau, mirroring the leveling of bulk properties and indicating saturation of entanglement density.

### Cyclic tensile behavior of RC hydrogels

**Figure 4:**
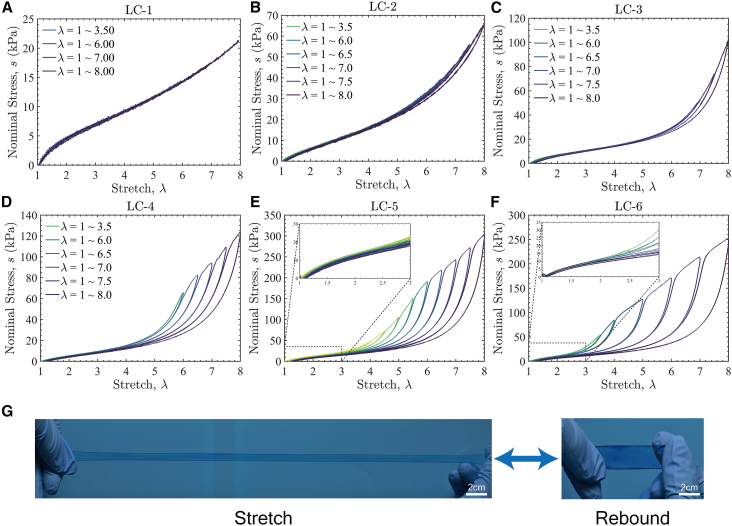
Cyclic stress-stretch response of RC hydrogels (A–F) Cyclic nominal stress-stretch curves of the LC series hydrogels. LC-1 to LC-6 exhibit negligible hysteresis at small to moderate stretches (*λ* < 3) but pronounced hysteresis at large stretches (*λ* ≥ 3). The initial modulus of RC hydrogels remains unaffected by large maximum history stretches, despite notable hysteresis during the cyclic tests. (G) Excellent rebound performance of LC-5. LC-5 repeatedly undergoes large deformations while maintaining elasticity and recoverability, without entering the plastic deformation regime.

The cyclic tensile behavior of RC hydrogels was further investigated. For the LC series hydrogels, as shown in Figures 4A–4F, the loading-unloading curves exhibit negligible hysteresis under small to moderate stretches (λ < 3), demonstrating excellent elastic recovery, as shown in Figure 4G. However, at large stretches, the hysteresis loop expands significantly, and this phenomenon becomes more pronounced as the number of interpenetrating networks increases. The cyclic tensile behavior of the MC and HC hydrogels, shown in Figure S8, exhibits behavior similar to that of the LC series hydrogels. At small to moderate stretches, negligible hysteresis is attributed to chain slippage at entanglement points within the interpenetrating network. These entanglement points function as slip-links, redistributing localized, concentrated stress to prevent excessive tension on individual chains and reducing the risk of chain rupture. However, under high stretches, the dense entanglement network and strong inter-chain interactions significantly constrain the motion of polymer chains, limiting slippage at entanglement points. This increased constraint causes some entanglements to become overstressed, leading to disentanglement and localized chain scission, which releases energy stored in the polymer chains. As more networks are incorporated through repeated crosslinking, the increasing entanglement density further restricts chain slippage, amplifying energy dissipation. Consequently, hydrogels with a higher degree of network entanglement (e.g., LC-5 and LC-6) exhibit more pronounced energy dissipation. Energy dissipation in conventional tough materials, such as filled rubber and double-network (DN) hydrogels, relies on the Mullins effect. Macroscopically, this effect manifests as stress softening and residual strain. This behavior is a consequence of irreversible damage to the primary load-bearing network. In contrast, the LC-5 and LC-6 materials exhibit no change in their initial modulus and residual strain, even after undergoing a large maximum pre-stretch. We attribute this exceptional stability to a distinct network architecture, created by our repeated crosslinking process, which generates numerous polymer chains that are only loosely entangled with the primary network. Under small-stretch deformation, these loosely entangled chains are not elastically active; as they are not part of the primary load-bearing network, they can slide relative to it without generating substantial retractive force, and thus contribute minimally to the material’s initial elastic modulus. At large stretches, however, these same chains behave as slip-links and undergo relative sliding, a process that dissipates substantial mechanical energy. Therefore, even if some of these loosely entangled chains rupture or become disentangled during large deformation, their impact on the initial modulus remains negligible, as this modulus is governed exclusively by the integrity of the primary network. The comparison of the cyclic tensile behavior of hydrogels with the same number of repeated crosslinking cycles but different crosslinking densities (e.g., LC-4, MC-4, and HC-4) reveals that the hysteresis loop area decreases with increasing crosslinking density. Higher crosslinking densities reduce the mean chain length, thereby limiting the stretchability of individual polymer chains. As a result, local chain rupture becomes more likely to trigger catastrophic failure and lead to fracture before significant hysteresis can develop.

### Fracture toughness and flaw sensitivity of RC hydrogels

**Figure 5:**
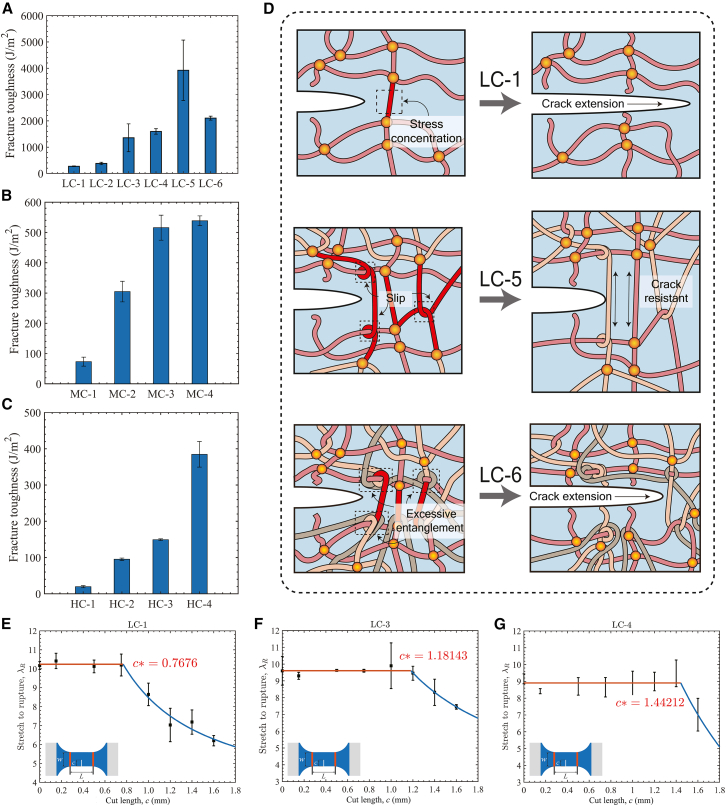
Fracture toughness and flaw sensitivity of RC hydrogels (A–C) Fracture toughness of the LC (A), MC (B), and HC (C) series hydrogels measured by pure shear tests. Data are presented as mean ± SD (*n* = 5). (D) Schematic illustration of the toughening mechanism in RC hydrogels. Different colors distinguish different polymer chain segments. In the early stages of repeated crosslinking (e.g., LC-5), entangled polymer chains enhance network uniformity and increase the number of chains restricting crack propagation, thereby enhancing fracture toughness. In the later stages (e.g., LC-6), excessive entanglement restricts chain orientation and deformation, increases the local stress concentration near the crack tip, and ultimately weakens the toughening effect. (E–G) Stretch to rupture *λ*_*R*_ as a function of cut length c for (E) LC-1, (F) LC-3, and (G) LC-4, showing a distinct transition from flaw-insensitive to flaw-sensitive behavior at the critical cut lengths *c*^∗^ of 0.7676 mm, 1.1814 mm, and 1.4421 mm, respectively. Data are presented as mean ± SD (*n* = 5).

The fracture toughness of RC hydrogels measured by pure shear tests is presented in Figures 5A–5C. Conventional single-network hydrogels, such as LC-1, exhibit a fracture toughness of only 272.28 J/m^2^, reflecting poor resistance to crack propagation. In LC-1 hydrogels, inherent structural heterogeneity often leads to localized stress concentration and chain rupture (indicated by red segments in Figure 5D, LC-1) at the crack tip, ultimately initiating crack propagation. However, with appropriate repeated crosslinking cycles, the newly formed network interpenetrates with the original network. Under external stretch, the slip mechanism between networks can transmit tension and reduce stress concentration around the crack tip. The number of effective polymer chains restricting crack propagation can therefore be increased, and these chains are oriented with external stretch, leading to an increase in fracture toughness (Figure 5D, LC-5). With excessive repeated crosslinking cycles, toughening mechanisms begin to diminish as the number of crosslinking cycles increases. This decline is attributed to the rising entanglement density within the polymer network. In highly entangled and rigid networks, excessive chain entanglement restricts polymer orientation and slippage near crack tips, preventing effective stress dissipation. As a result, chains rupture prematurely at stress concentration sites, leading to reduced fracture toughness (Figure 5D, LC-6). This behavior also suggests that an optimal number of crosslinking cycles exists to achieve maximum toughness enhancement.

The flaw sensitivity of RC hydrogels is evaluated to assess the reduction in stretchability caused by cuts and flaws.^45^ It can be measured through uniaxial tensile tests on dumbbell-shaped specimens with varying cut lengths (*c*) (Figures 5E–5G). Here, we measured the flaw-sensitivity of the LC series hydrogels, and the experimental results are shown in Figures 5E–5G. For all test hydrogels, there exists a critical length (*c*^∗^), below which the stretch to rupture (*λ*_R_) remains nearly constant. However, when the cut length exceeds *c*^∗^, *λ*_R_ decreases rapidly with increasing cut length. As reported by Chen et al.,^45^ this difference results from a shift in the dominant fracture mechanism from stress concentration to stress blunting at the crack tip as the cut length decreases. The results shown in Figures 5E–5G indicate that *c*^∗^ increases from 0.7676 mm to 1.4421 mm as the number of repeated crosslinking cycles increases. The enhanced crack resistance of the hydrogel arises from polymeric entanglement networks formed through repeated crosslinking. Conventional SN hydrogels, which undergo only a single crosslinking step, often develop microstructural heterogeneities that serve as stress concentrators at crack tips during deformation, promoting premature scission of nearby polymer chains. Once a chain segment ruptures, the stress rapidly transfers to adjacent chains, initiating a progressive chain scission process that accelerates crack propagation and results in brittle, defect-sensitive failure. In contrast, RC hydrogels feature a densely entangled network with improved homogeneity that redistributes external stress and reduces crack tip stress concentration. Interpenetrating chains accommodate deformation through slippage and progressive rupture within the high-strain zone, dissipating fracture energy, and retarding crack growth. By spatially distributing energy dissipation across the network, this mechanism enhances defect tolerance and prevents catastrophic failure.

**Figure 6:**
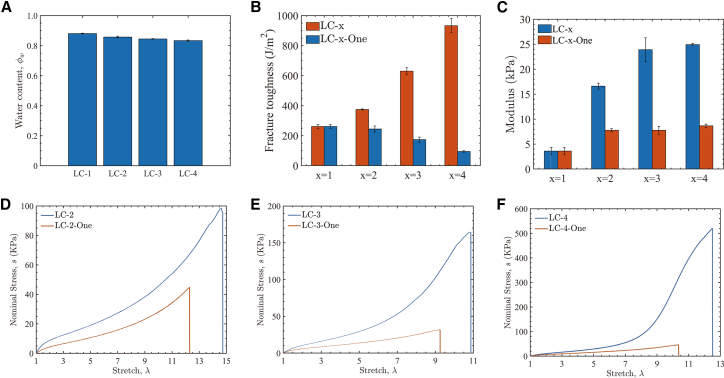
Comparison of mechanical properties between RC and SN hydrogels with the same water content (A) The water content of RC series LC-x measured by freeze-drying shows a slight decrease with the number of crosslinking cycles. (B and C) Fracture toughness and initial elastic modulus for RC (LC-x) hydrogels compared to SN controls (LC-x-One) at matched water content. LC-x denotes an RC hydrogel after x crosslinking cycles; LC-x-One denotes an SN control synthesized with identical feed ratios and individually matched water content. Data are presented as mean ± SD (*n* = 5). (D–F) Nominal stress–stretch (*σ*– *λ*) curves for RC hydrogels and SN controls at matched water content. RC hydrogels exhibit higher tensile strength and stretchability compared with the SN hydrogels.

As the number of repeated crosslinking cycles increases, the overall water content of the RC hydrogels decreases slightly (Figure 6A). To eliminate the influence of water content on mechanical properties and confirm that the toughening of RC hydrogels originates from network evolution induced by repeated crosslinking, we prepared a series of SN control samples. Each SN hydrogel was synthesized using the same monomer, crosslinker, and initiator molar ratios as its RC counterpart, and its water content was individually matched to that of the corresponding RC sample. For example, RC hydrogel sample LC-4 (after four crosslinking cycles) had a water content of 84.56%, as determined after freeze-drying. We therefore prepared an SN control sample with the same water content, designated LC-4-One. As shown in Figures 6B and 6C, at equal water content, the RC hydrogels exhibit significantly higher fracture toughness and initial elastic modulus than the corresponding SN controls, and this performance gap widens further with each additional crosslinking cycle. In addition, uniaxial tensile stress-stretch curves (Figures 6D–6F) show that the RC hydrogels have a higher tensile strength and a greater elongation at break than their SN counterparts. These results clearly demonstrate that the enhanced mechanical properties of the RC hydrogels are not due to water content differences, but rather to the progressive refinement and homogenization of the polymer network by repeated crosslinking. Specifically, the repeated crosslinking process increases the effective chain density and fills network defects such as voids and free chain ends, which mitigates local stress concentrations. In addition, at large strains, chain entanglements serve as physical cross-links that allow polymer segments to slide past one another, enabling more efficient energy dissipation. Therefore, even at equal water content, the RC hydrogels still exhibit substantially higher fracture toughness and load-bearing capacity than the SN controls.

### Fatigue and wear resistance of RC hydrogels

**Figure 7:**
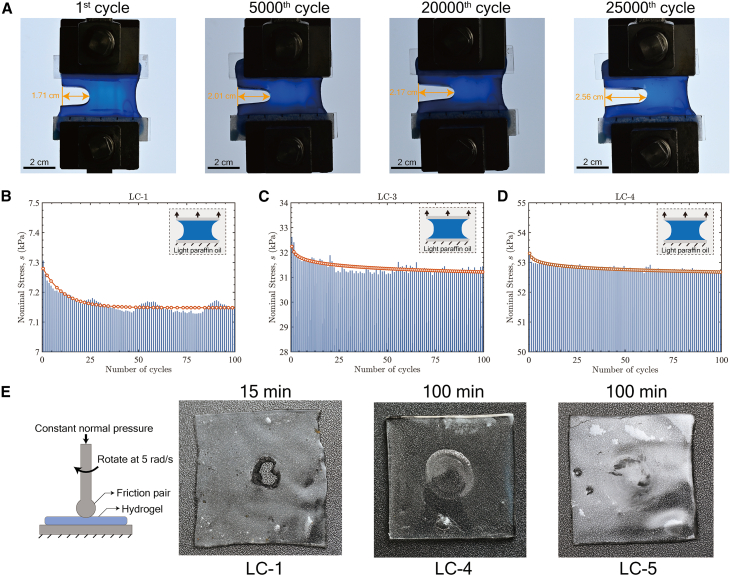
Fatigue and wear resistance of RC hydrogels (A) Photographs of crack evolution in LC-5 with an initial cut length of 20 mm under cyclic loading (*λ* = 1.0–2.0, 5 Hz), showing gradual crack extension from 1.71 cm to 2.56 cm after 25,000 cycles. (B–D) Stress evolution of (B) LC-1, (C) LC-3, and (D) LC-4 during cyclic loading (*λ* = 1.0–6.0, 0.1 Hz). (E) Wear-resistance test of RC hydrogels. Under constant pressure, LC-1 hydrogel ruptured after 15 min, whereas LC-4 and LC-5 remained intact after 100 min.

Fatigue resistance under cyclic loading is a critical performance criterion for soft polymeric materials in engineering applications. To evaluate the fatigue resistance of RC hydrogels, fatigue crack propagation experiments were performed. Rectangular hydrogel specimens (50 mm × 10 mm) with a 20 mm edge crack were subjected to cyclic loading using a triangular waveform at 5 Hz, with the stretch ratio (*λ*) ranging from 1 to 2. In order to ensure that the water content of the test sample remains constant, all samples were fully immersed in light paraffin oil within a custom-designed acrylic chamber. For crack propagation visualization, hydrogels were pre-stained with methylene blue, and images were captured at maximum stretch (*λ* = 2). Experimental results indicate that LC-5 exhibited minimal crack growth—from 1.71 cm to 2.01 cm—after 5000 loading cycles (Figure 7A), corresponding to a crack growth rate of d*a*/d*N* = 0.102 μm/cycle. In contrast, under identical loading conditions, SN hydrogels (LC-1) showed substantial crack growth from 1.65 cm to 3.19 cm, yielding a markedly higher crack growth rate of *da*/*dN* = 6.0 μm/cycle (Figure S9). These results demonstrate that the repeated crosslinking strategy effectively mitigates stress concentration at the crack tip and facilitates energy dissipation, thereby enhancing fatigue fracture resistance. To further assess fatigue damage performance, we measured the evolution of maximum stress in uncracked RC hydrogels subjected to cyclic tensile tests. Samples with identical dimensions were immersed in light paraffin oil and cyclically stretched from *λ* = 1 to 6 at 0.1 Hz. As shown in Figures 7B–7D, the maximum stress decreased by approximately 2.05% in LC-1, 1.24% in LC-3, and only 0.377% in LC-4 after 100 cycles. This performance enhancement is attributed to improved network uniformity in RC hydrogels, which compensates for pre-existing structural defects and reduces localized strain concentrations, thereby delaying damage initiation and suppressing stress degradation during cyclic loading, finally improving fatigue damage resistance. The wear resistance of RC hydrogels is also evaluated. As shown in Figure 7E, a constant normal pressure of 100 kPa was applied to hydrogel samples with different crosslinking cycles while the friction pair rotates at an angular velocity of 5 rad/s. The combination of high toughness and high effective chain density results in a high wear resistance of RC hydrogels. The LC-1 hydrogel fractured after 15 min of testing, whereas the LC-4 and LC-5 hydrogels remained intact even after 100 min of testing.

### Large deformation sensor based on RC hydrogel

**Figure 8:**
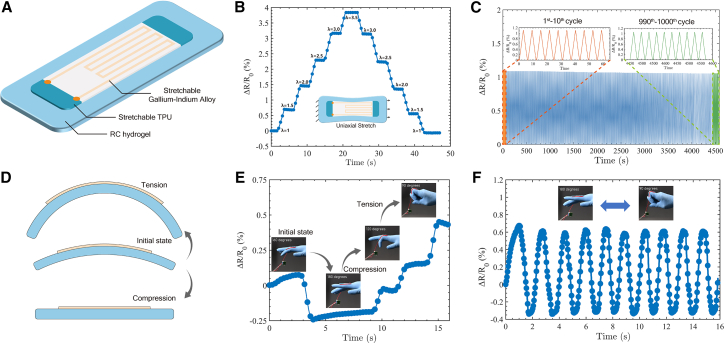
Hydrogel-integrated large-deformation sensor (A) Sensor architecture: eutectic gallium-indium (EGaIn) conductor patterned on a stretchable TPU film and bonded to an RC-hydrogel substrate. (B) ΔR/R0 versus stretch (*λ*) during stepped uniaxial loading–unloading, showing an approximately linear response with minimal hysteresis within the tested range (λ≤3.5). (C) Cyclic durability: ΔR/R0 remains stable over 1,000 cycles at $\lambda_{\max}=3.5\text{.}$ Insets compare cycles 1–10 and 990–1,000. (D) Bending-sensing mechanism: off-neutral-axis placement yields tensile strain on the convex side and compressive strain on the concave side, producing opposite resistance changes. (E) Real-time ΔR/R0 trace of the sensor from neutral to compression and back to tension during the finger motion sequence. (F) Repeatable signals over multiple flexion-extension cycles of the sensor during finger bending.

The synergistically enhanced mechanical properties of the RC hydrogel make it an effective substrate for robust, large-deformation strain sensors. This combination of high strength, toughness, fatigue resistance, and stretchability provides the durability and broad strain range required for wearable sensing. Furthermore, negligible hysteresis at small-to-moderate strains ensures minimal signal drift, long-term stability, and reliable, conformal contact, which are key requirements for high-fidelity sensors. Together, these attributes position the RC hydrogel as a promising platform for precise human-motion tracking. Based on the excellent mechanical properties of RC hydrogel, we fabricated a flexible strain sensor by laminating a thermoplastic polyurethane (TPU) film encapsulating an eutectic gallium-indium (EGaIn) circuit onto an LC-5 substrate (Figure 8A). The sensor operates by geometric deformation of the liquid-metal trace: substrate strain stretches and narrows the EGaIn pathway, increasing its electrical resistance. Calibration shows a monotonic, approximately linear dependence of ΔR/R_0_ on applied stretch (*λ*), enabling quantitative strain readout (Figure 8B). The device exhibited stable performance over extended cycling, attributable to the exceptional mechanical performance of the RC-hydrogel substrate. Within the operating window (λ<3.5), the small ΔR/R_0_ hysteresis area suppresses baseline drift, and the substrate’s intrinsic fatigue resistance preserves signal integrity during repeated use. Cyclic tests confirmed this robustness: the electrical response remained consistent and repeatable over 1,000 stretching cycles (Figure 8C). The high stretchability and soft, compliant interface of the sensor make it suitable for human-motion monitoring. The bending-sensing mechanism (Figure 8D) uses off-neutral-axis placement—bending puts the sensing layer in tension or compression, which increases or decreases resistance, respectively. To evaluate on-skin performance, we attached the sensor to the index-finger interphalangeal joint (Figures 8E and 8F). The device tracked joint motion in real time during continuous flexion-extension, with ΔR/R_0_ reproducing the gesture dynamics (The whole joint movement monitoring can be seen in the Supporting Video S2).

## Discussion

In summary, this study presents a universal strategy for simultaneously enhancing multiple mechanical properties of hydrogels through successive rounds of crosslinking within a single polymer network. Using polyacrylamide hydrogels as a model system, five crosslinking cycles yield RC hydrogels (e.g., LC-5) with nearly a 6-fold increase in elastic modulus, a 10-fold increase in fracture toughness, a 20-fold increase in tensile strength, and a 42-fold increase in work of fracture compared to their SN counterparts (e.g., LC-1), while maintaining high stretchability and minimal hysteresis under stretches up to *λ* = 3. In addition, RC hydrogels demonstrate lower flaw sensitivity, greater fatigue resistance, and superior puncture and wear resistance. These substantial improvements arise from the relatively uniform network architecture formed by the repeated crosslinking of polymer networks, enriched with polymer chain entanglements that act as slip-links. Under small to moderate deformation, the entangled network retains elasticity and structural integrity. At large stretches, however, the disengagement of slip-links and breakage of polymer chains introduced by later network crosslinking dissipate energy and delay crack propagation without reducing the initial elastic modulus. In this way, RC hydrogels overcome traditional trade-offs in hydrogel design without the need for specialized chemical components or complex processing. This straightforward yet effective approach offers a robust platform for fabricating hydrogels with comprehensively enhanced mechanical performance, paving the way for their use in load-bearing and wear-resistant soft tissue replacement, flexible electronic substrate, cardiac healing patch, and other demanding soft-matter applications.

### Limitations of the study

In this study, the mechanical reinforcement of hydrogels achieved through the repeated crosslinking strategy was primarily demonstrated using PAAm hydrogels at specific formulations. While this strategy is theoretically applicable to other hydrogel compositions or broader classes of soft materials, the specific numerical improvements reported here may vary across different material systems. Furthermore, although we proposed a plausible reinforcement mechanism based on comprehensive macromechanical and microstructural characterizations, direct real-time visualization or atomic-scale verification of this mechanism remains challenging. Future advancements in high-resolution, *in-situ* experimental techniques will be essential to further validate and refine these findings.

## Resource availability

### Lead contact

Further information and requests for resources and reagents should be directed to and will be fulfilled by the lead contact, Zishun Liu (zishun.liu@cityu-dg.edu.cn).

### Materials availability

This study did not generate new unique reagents.

### Data and code availability

• All data reported in this paper will be shared by the lead contact upon request.
• This paper does not report original code.
• Any additional information required to reanalyze the data reported in this paper is available from the lead contact upon request.

## Acknowledgments

The authors are grateful for the support from the 10.13039/501100001809National Natural Science Foundation of China (no. 12172273 and 12202339). L.Z.S. and Z.Z.D. also acknowledge support from the Guangdong and Hong Kong Universities “1+1+1” Joint Research Collaboration Scheme. Part of this work was facilitated by the utilization of the advanced electron microscopy facilities housed within the XJTU-NIN-Hitachi Joint Research Center and the state key laboratory of electrical insulation and power equipment at Xi’an Jiaotong University, China. We would like to acknowledge Chuanwei Fan for his assistance with microscope operation, data processing, and valuable discussions.

## Author contributions

Conceptualization: H.L. and Z.Z. methodology: H.L. and Z.Z. validation: H.L., Z.Z., Y.G., J.L., and Z.L. data curation: Y.G., supervision: J.L. and Z.L. writing – original draft: H.L. and Z.Z. writing – review and editing: H.L., Z.Z., J.L., and Z.L.

## Declaration of interests

The authors declare no competing interests.

## STAR★Methods

### Key resources table

**Table:** 

REAGENT or RESOURCE	SOURCE	IDENTIFIER
**Chemicals, peptides, and recombinant proteins**		

ACRYLAMIDE	SHANGHAI MACKLIN BIOCHEMICAL CO., LTD. (SHANGHAI, CHINA)	CAS: 79-06-1
N, N′-METHYLENEBISACRYLAMIDE	ALADDIN BIOCHEMICAL TECHNOLOGY CO., LTD (SHANGHAI, CHINA)	CAS: 110-26-9; PubChem CID: 8041
Α-KETOGLUTARIC ACID	ALADDIN BIOCHEMICAL TECHNOLOGY CO., LTD (SHANGHAI, CHINA)	CAS: 328-50-7

**Software and algorithms**		

MATLAB	MATLAB 2022A	https://www.mathworks.com

### Experimental model and study participant details

Omitted as our study does not involve biological models.

### Method details

#### Material synthesis

RC hydrogels were synthesized using a multi-step polymerization method. In each polymerization step, acrylamide (AAm; Macklin), N, N′-methylenebisacrylamide (MBAA; Aladdin), and α-ketoglutaric acid (α-Keto; Aladdin) were used as the monomer, crosslinker, and radical initiator, respectively. The precursor solution for the LC hydrogel with x number of networks (LC-x) was prepared by first dissolving 12.2 g of AAm, 0.0123 g of MBAA, and 0.0292 g of α-ketoglutaric acid in 100 mL of deionized water. The precursor solution was then poured into a glass mold and exposed to UV light (80 W, 365 nm) for 10 h to initiate polymerization, resulting in the formation of an SN hydrogel, referred to as LC-1. Once LC-1 was formed, it was immersed in a large volume of the same precursor solution for at least 24 h to fully swell. After which, the hydrogel was placed between two glass plates and cured again under UV light to form the second network, referred to as LC-2. By repeating the swelling and curing cycle, the LC-x can be synthesized. For the preparation of MC and HC hydrogels, the process was identical, with the exception of the amount of MBAA used. Specifically, the MBAA dosages were adjusted to 0.0246 g and 0.0492 g for the MC and HC hydrogels, respectively, while the amounts of AAm, α-Keto, and deionized water remained constant. The crosslinker-to-monomer molar ratios for the LC, MC, and HC precursor solutions were calculated to be 0.00046, 0.00093, and 0.00186, respectively. SN hydrogels with the same molar ratio of monomer to crosslinker and initiator, as well as the same water content as the RC hydrogels LC-2, LC-3, and LC-4, are labeled LC-2-One, LC-3-One, and LC-4-One, respectively. These hydrogels were synthesized by first measuring the water content of the LC-2/3/4 hydrogels using freeze-drying. Then, based on this water content, the feed concentration for the synthesis of the SN counterpart was obtained. For example, the water content of LC-2 was measured to be 86.28%. Based on this value, the amounts of deionized water, AAm, MBAA, and α-keto required to cure the LC-2-One were calculated to be 76.9823 g, 12.2 g, 0.0123 g, and 0.0292 g, respectively.

#### Uniaxial tensile tests

In this study, uniaxial tensile tests were performed under both monotonic and cyclic loading modes to evaluate the stress-stretch curves and hysteresis of hydrogels. Standard dumbbell-shaped specimens (gauge length 20 mm, width 4 mm) were stretched at a velocity of 50 mm/min using a universal testing machine (SHIMADZU AGS-X, 100-N load cell). Nominal stress was calculated from the applied force *F* divided by the initial cross-sectional area of the specimen *A*_0_ (*σ* = *F*/*A*_0_), and stretch was determined by the ratio of the current measured length *L* to the initial gauge length *L*_0_ (*λ* = *L*/*L*_0_). The stress-stretch curves of hydrogels exhibit a typical two-stage behavior: strain softening at small stretches and pronounced strain hardening at large stretches. The initial elastic modulus of the hydrogel was obtained by linear fitting of the nominal stress-stretch data within the 3% strain range.

#### Puncture tests

The puncture test was performed using a universal tensile testing machine (SHIMADZU AGS-X, 100-N load cell). A round-shape hydrogel specimen with a radius of 10 cm and thickness of 1.8 mm was securely fixed onto a custom holder. The holder was mounted on the tensile testing machine, and a spike with a diameter of 10 mm was attached to the upper crosshead. The spike was moved downward at a constant velocity of 50 mm/s, applying force to the hydrogel film until penetration occurred. The maximum allowed displacement of the apparatus is limited to 100 mm.

#### Wear tests

The wear resistance of the hydrogel was evaluated using an HT-500 friction and wear tester (ZHONGKEKAIHUA). A hydrogel specimen was cut into a square shape with a side length of 3.0 cm and a thickness of 1.8 mm. A normal pressure of 100 kPa was applied to the hydrogel specimen. The rotation speed of the friction pair was set to 5 rad/s and maintained in continuous contact with the hydrogel. During the test, the temperature of the hydrogel was controlled at 35 °C.

#### Scanning electron microscopy (SEM) characterization

To observe the microstructure of hydrogels, hydrogel samples were prepared using a low-temperature brittle-fracture technique. First, pristine hydrogel specimens were rapidly immersed in liquid nitrogen to freeze and preserve their three-dimensional network structure. Next, the frozen specimens were placed in a freeze dryer at −50°C under vacuum conditions for 120 h until they reached a constant weight (defined as less than a 0.5% variation between two consecutive weighing). After freeze-drying, the specimens were immersed in liquid nitrogen again for a deep-freezing treatment, ensuring the hydrogel remained in a glassy state. Brittle fracture surfaces were then created using the three-point bending method, making them suitable for SEM observation. To eliminate charging effects during imaging, the fracture surfaces were sputter-coated with gold using an ion sputtering apparatus (15 mA sputtering current, 60 s duration). Finally, the microstructure of the fracture surfaces was examined using a field-emission SEM (CIQTEK FESEM4000) at an acceleration voltage of 3 kV and a working distance of 10 mm.

#### Pure shear tests

Fracture toughness of the hydrogel was measured by pure shear tests conducted on a universal testing machine (SHIMADZU AGS-X, 100-N load cell). Specimens with and without 20 mm pre-cracks were prepared using rectangular dies (50 mm × 30 mm). The obtained specimens were stretched at a loading speed of 50 mm/min until fracture occurred. The critical stretch at which the pre-crack propagates determines *λ*_f_. To accurately capture this critical stretch, the crack tip was stained with methylene blue and imaged using a high-speed camera. The fracture toughness (*Γ*) of the hydrogel was calculated by integrating the nominal stress-stretch curve of specimens without pre-cracks, from 1 to *λ*_f_:

(Equation 6) $$\begin{array}{c} \Gamma =H\int_{1}^{\lambda_{f}}\sigma \left(\lambda \right)d\lambda \end{array}$$

where *H* represents the initial height of the testing region in the specimen (10 mm), *σ* denotes the nominal stress, and *λ* is the stretch.

#### Fatigue tests

Fatigue tests were conducted using hydrogel specimens with dimensions identical to those used in pure-shear tests (50 mm × 30 mm) on a universal testing machine (SHIMADZU AGS-X, 100-N load cell). To prevent dehydration of hydrogels during the extended duration of fatigue tests, each test specimen was fully immersed in light paraffin oil within a custom-designed acrylic chamber (as shown in Figure S10. Specimens without pre-cracks were cyclically stretched to a displacement of 50 mm at a loading rate of 500 mm/min, with the peak stress evolution continuously monitored throughout the entire testing process. For specimens with pre-cracks, cyclic stretching was carried out at the same rate to a displacement of 10 mm, with crack propagation observed and recorded after every 1,000 cycles. As the paraffin oil film formed on the acrylic box surfaces interfered with imaging quality, specimens with pre-cracks were removed from the oil bath at each 1,000-cycle interval and subsequently re-stretched at the same loading rate to a displacement of 10 mm to clearly document the crack propagation process.

#### The measurement of water content

The water content of hydrogels was quantitatively determined by freeze-drying. Hydrogel samples with an initial mass *m*_*i*,*a*_ were freeze-dried at −50°C until they reached a constant weight *m*_*i*,*b*_. The water content (*W*_i_) was then calculated using the following equation:

(Equation 7) $$\begin{array}{c} W_{i}=1-\frac{m_{i,b}}{m_{i,a}} \end{array}$$

#### Quantifying network pre-stretch and polymer weight fraction

To characterize the hydrogels synthesized via *K* sequential crosslinking cycles (LC-K), we measured the total weight (*m*_*i*_) and thickness (*t*_*i*_) after each cycle *i*. Based on these data, we first derived the swelling-induced pre-stretch ratio of the *i*^*th*^ network *λ*_0,*i*_ through:

(Equation 8) $$\begin{array}{c} \lambda_{0,i}=\frac{t_{K}}{t_{i}} \end{array}$$

This ratio quantifies the pre-stretch imposed on the *i*^*th*^ network within a hydrogel with a thickness of *t*_*i*_ relative to its state within the final, fully swollen hydrogel (with a thickness *t*_*K*_). Next, we defined the polymer weight fraction, *ϕ*_*i*_, of the *i*^*th*^ network. We first determine the polymer mass after the *i*^*th*^ cycle, *m*_*p*,*i*_, using the measured total mass (*m*_*i*_) and water content (*W*_*i*_, mass fraction): *m*_*p*,*i*_ = (1-*W*_*i*_)*m*_*i*_. The parameter *ϕ*_*i*_ is then calculated as the ratio of the polymer mass added during the *i*^*th*^ cycle (*m*_*p*,*i*_ − *m*_*p*,*i*-1_) to the final total polymer mass (*m*_*p*,*K*_):

(Equation 9) $$\begin{array}{c} \phi_{i}=\frac{m_{p,i}-m_{p,i-1}}{m_{p,K}}=\frac{\left(1-W_{i}\right)m_{i}-\left(1-W_{i-1}\right)m_{i-1}}{\left(1-W_{K}\right)m_{K}} \end{array}$$

Here, *W*_*K*_ and *m*_*K*_ are the water content and total weight, respectively, of the final LC-K hydrogel. For the initial cycle (*i* = 1), *W*_0_ = 0 and *m*_0_ = 0 are applied.

### Quantification and statistical analysis

All quantitative results are reported as mean ± standard deviation (SD), and error bars in every figure denote ±SD. The number of independent specimens (n) per condition is specified in each figure legend. Curve fitting, data analysis, and visualization were performed in MATLAB R2022a (MathWorks). No inferential hypothesis testing (e.g., Student’s *t* test or ANOVA) was applied, as the comparisons in this study describe physical-property trends across material conditions rather than statistical contrasts among biological replicates; consequently, no significance asterisks appear in any figure.

## Supplemental information

Document S1. Figures S1–S15, Tables S1 and S2, and Methods S1

Video S1. Includes puncture and load-bearing tests of the LC series hydrogels (corresponding to Figures 1 and 2)

Video S2. Includes demonstration of the hydrogel-integrated large-deformation sensor to monitor joint movement
