# Supplementary material for: Comprehensive mechanical reinforcement of hydrogels via network refinement
Source: iScience. 2026 Jun 1;29(6):116191. doi: 10.1016/j.isci.2026.116191 (PMC13233583; doi:10.1016/j.isci.2026.116191)
Supplement: Document S1. Figures S1–S15, Tables S1 and S2, and Methods S1 [file mmc1.pdf]

iScience, Volume *29*

Supplemental information

Comprehensive mechanical reinforcement of hydrogels via network refinement

Han Li, Zidi Zhou, Yuan Gao, Jincheng Lei, and Zishun Liu

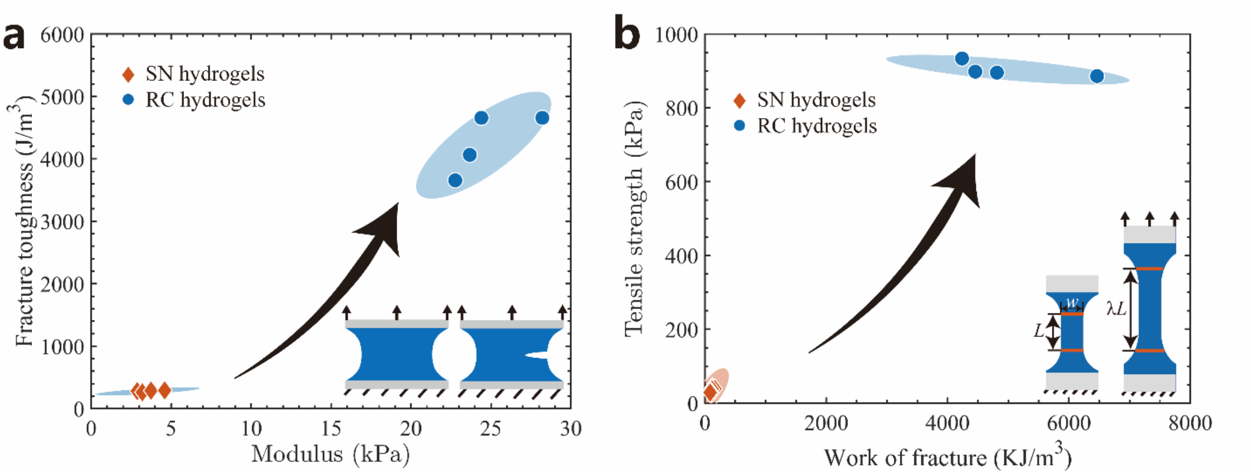

Figure S1. Comparison between regular SN hydrogels and RC hydrogels.
(a) Fracture toughness and initial elastic modulus of RC hydrogels and their counterpart SN hydrogels. **(b)** Tensile strength and work of fracture of RC hydrogels and their counterpart SN hydrogels.

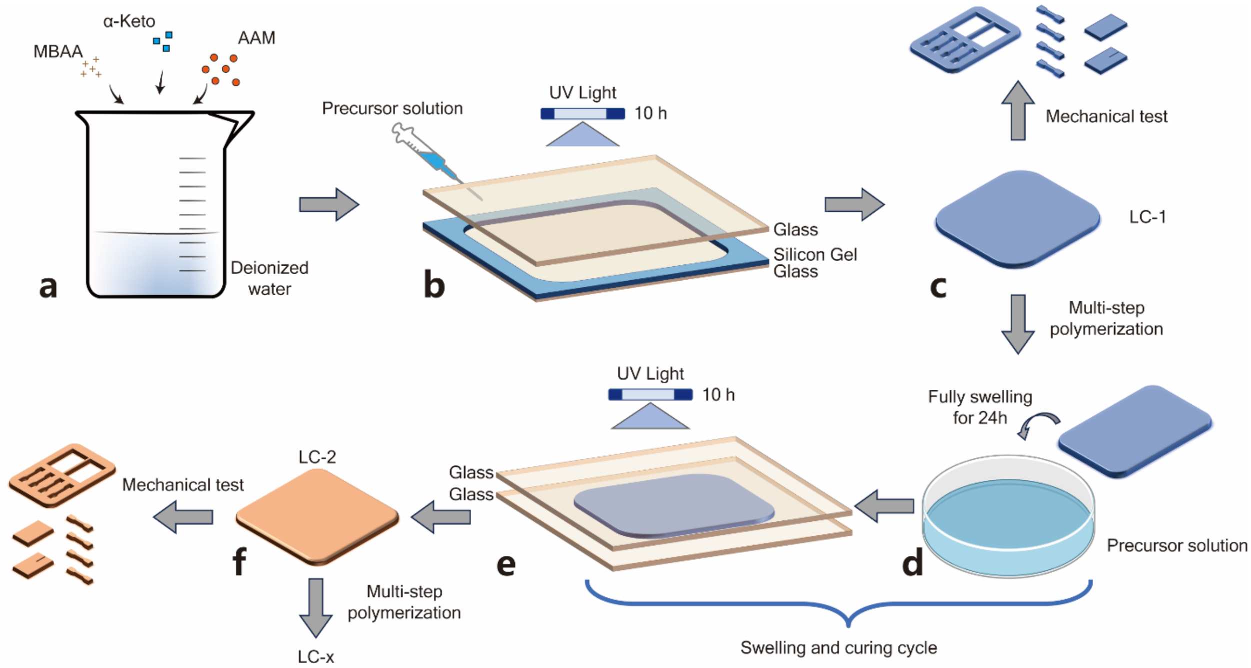

Figure S2. Fabrication procedure for RC hydrogels, illustrated using the LC-x series as an example. **(a)** Preparation of precursor solution containing a radical initiator. **(b)** Inject the precursor solution into a mold formed by two parallel glass plates separated by silicone spacers and cure under UV irradiation (365 nm, 80 W) for 10 hours. **(c)** Remove the glass plates and silicone spacers to obtain the SN hydrogel (LC-1). **(d)** Immerse the LC-1 samples in the same precursor solution for 24 hours to allow full swelling of the hydrogels. **(e)** Put the swollen hydrogel between glass plates again and perform a second UV curing step for an additional 10 hours to form the RC hydrogel (LC-2). **(f)** Repeat the swelling–curing cycle (x–1) times to fabricate LC-x.

Table S1. Formulation of the precursor solution for LC, MC, and HC hydrogels.

Hydrogels	AAm [g]	MBAA [g]	α-Keto [g]	Deionized water [ml]
LC	12.2	0.0123	0.0292	100
MC	12.2	0.0246	0.0292	100
HC	12.2	0.0492	0.0292	100

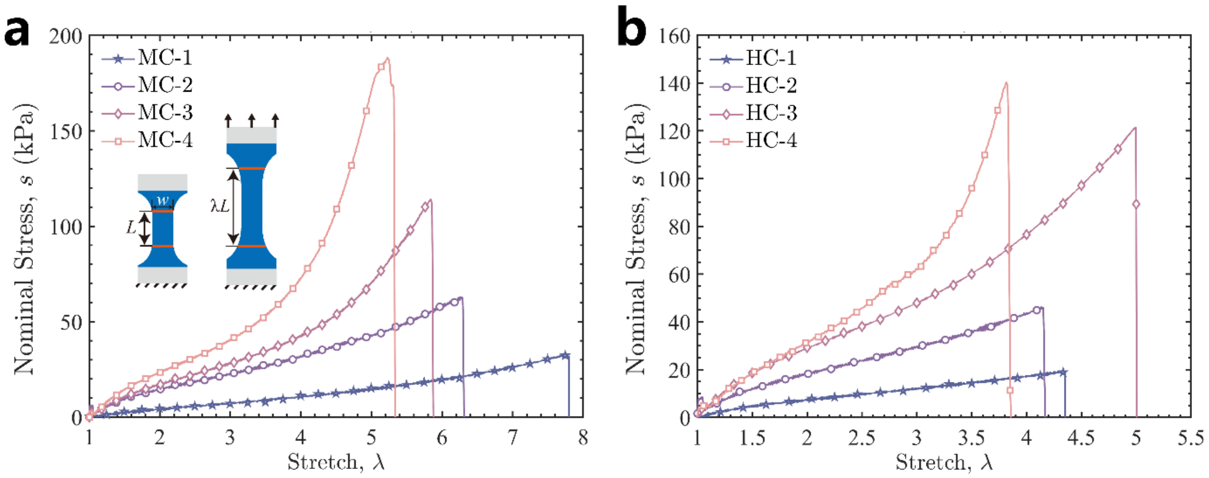

Figure S3. Nominal stress-stretch curves of (a) MC and (b) HC series hydrogels.

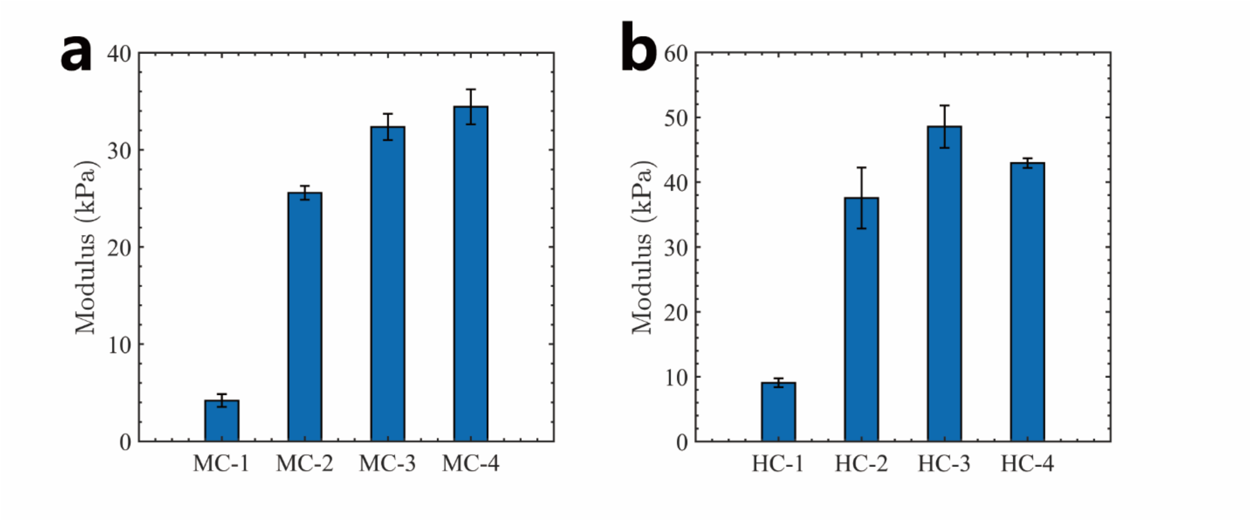

Figure S4. Initial elastic modulus of (a) MC and (b) HC series hydrogels.

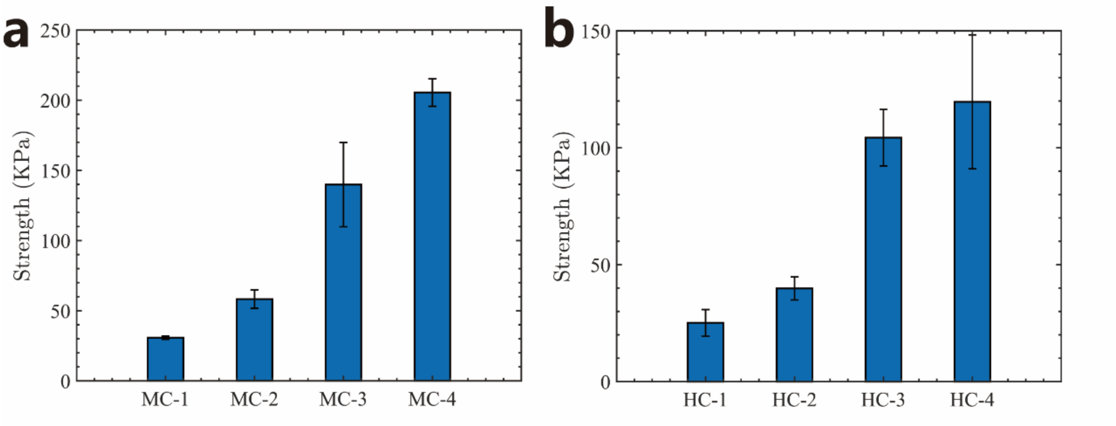

Figure S5. Uniaxial tensile strength of (a) MC and (b) HC series hydrogels.

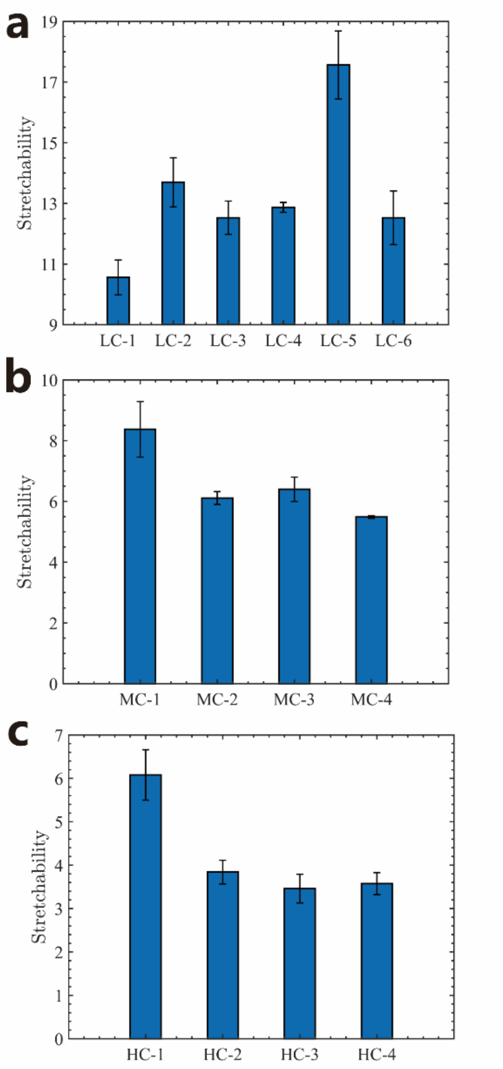

Figure S6. Stretchability of LC, MC, and HC series hydrogels with varying numbers of crosslinking cycles under uniaxial tensile tests.

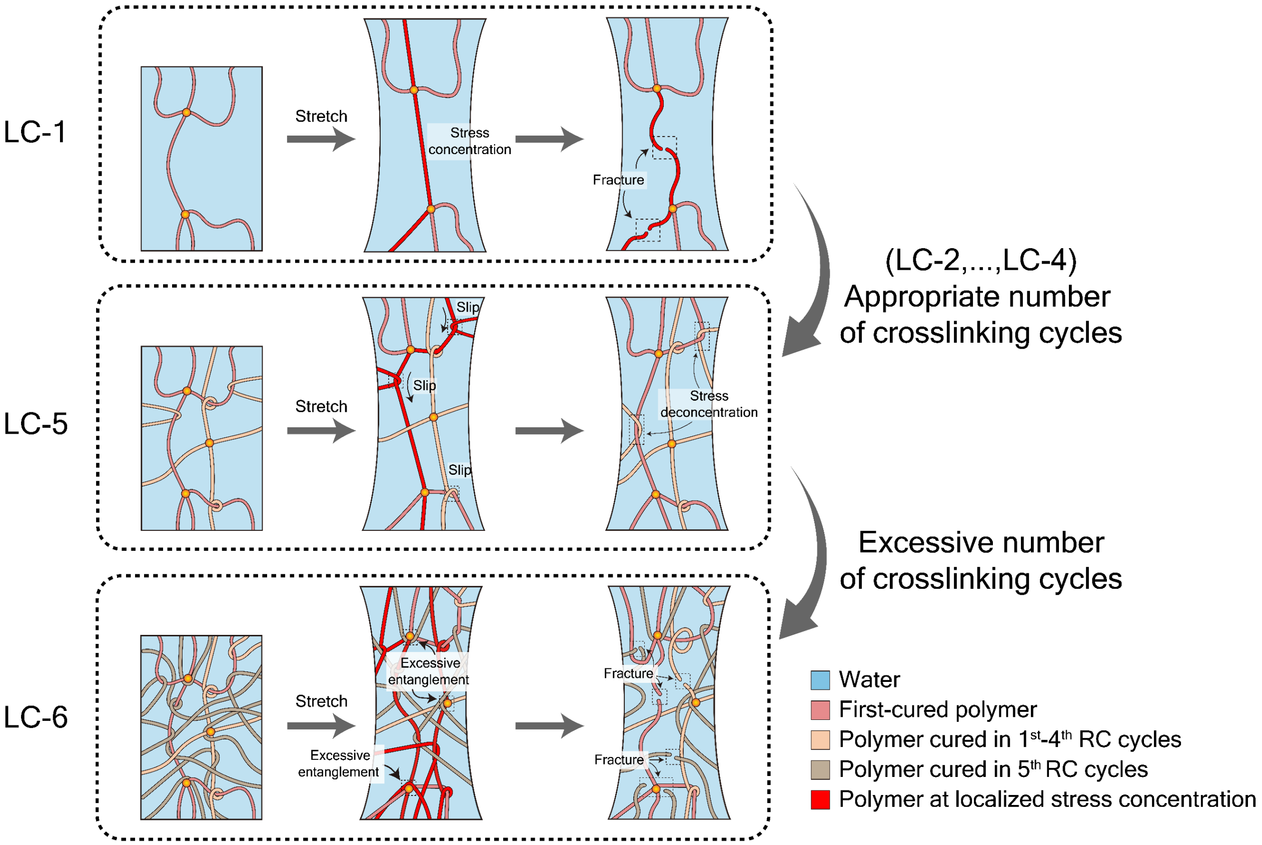

Figure S7. Schematic illustration of the toughening mechanism in RC hydrogels with different numbers of crosslinking cycles.

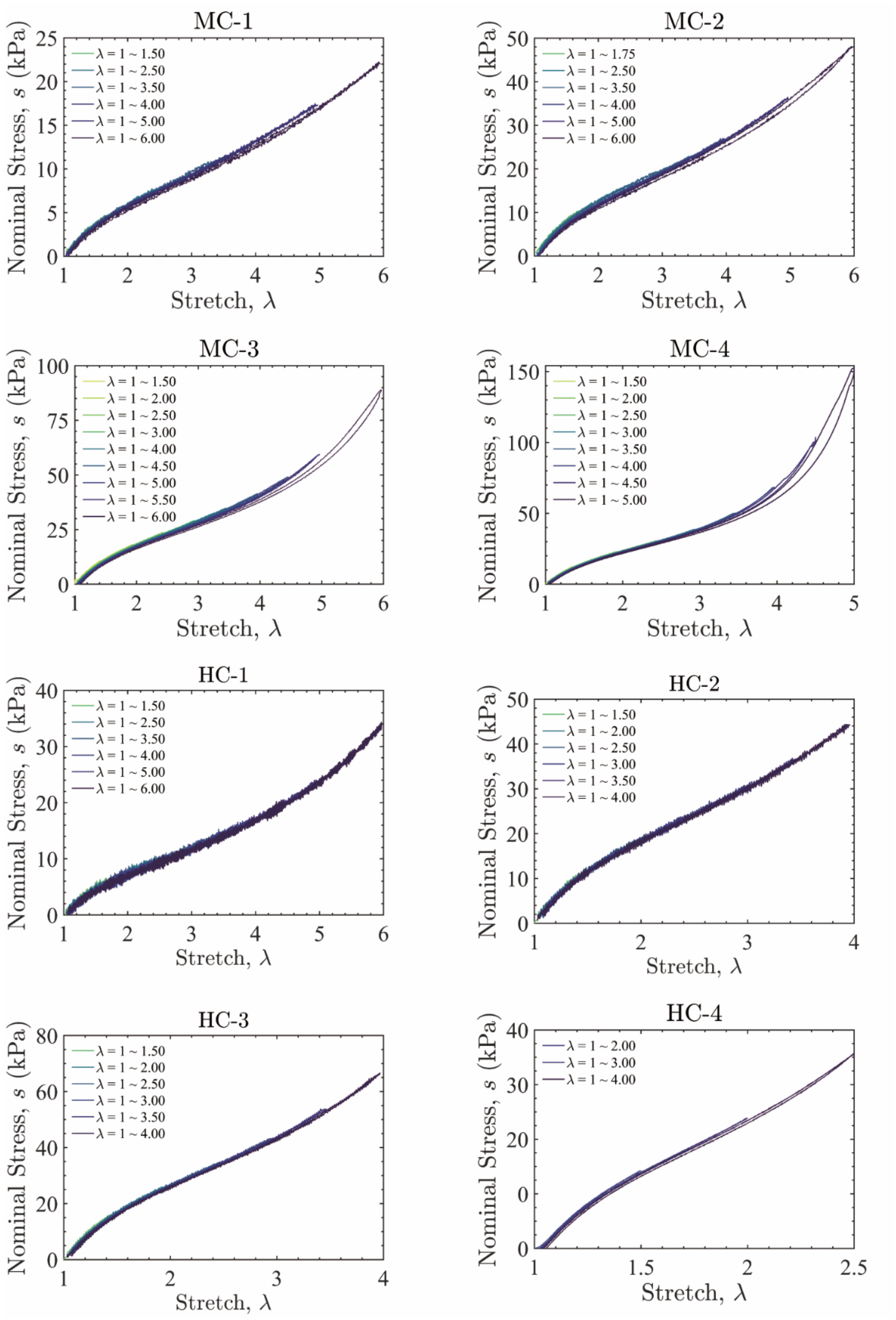

Figure S8. Nominal stress-stretch behaviors of MC and HC hydrogels under cyclic uniaxial tensile loading.

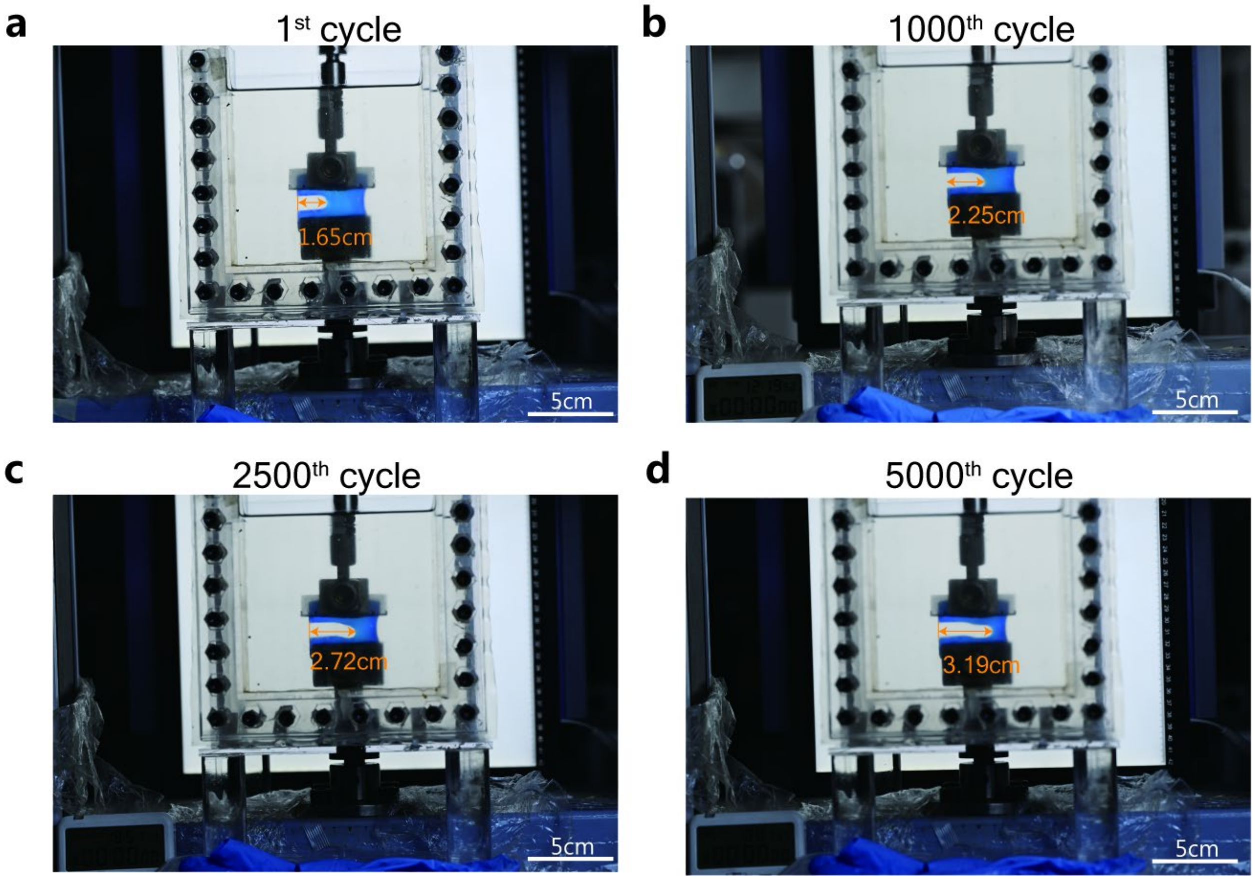

Figure S9. Fatigue crack propagation in LC-1 hydrogel.
The crack length progressively increased from **(a)** 1.65 mm at the 1st cycle to **(b)** 2.25 mm at the 1000th cycle, **(c)** 2.72 mm at the 2500th cycle, and (d) 3.19 mm at the 5000th cycle, at the maximum displacement of 10 mm.

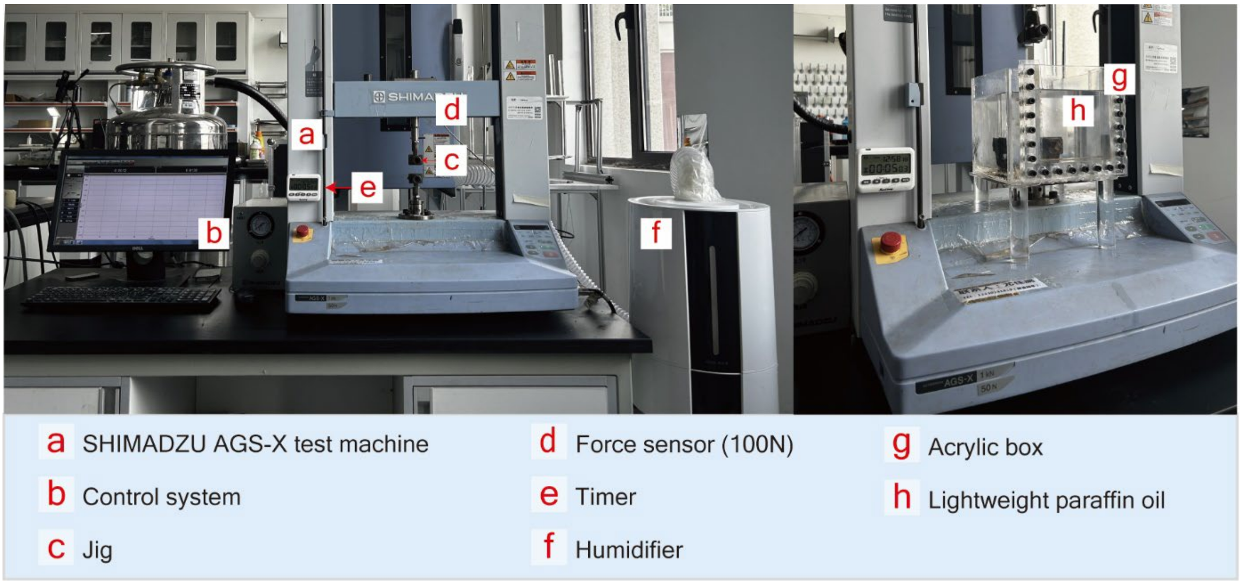

Figure S10. Mechanical testing apparatus for hydrogels.
A humidifier ensures constant humidity around the test specimen during the monotonic loading tests to maintain stable hydrogel water content. For cyclic loading tests, specimens were immersed in light paraffin oil within a custom-designed acrylic box to prevent water evaporation.

METHODS S1. MODELING AND SIMULATION DETAILS FOR THE MESOSCOPIC NETWORK MODEL, RELATED TO STAR METHODS.

Construction of the Mesoscopic Network Model

Using the mesoscopic network model, both uniform and random network model was developed. The network consists of discrete nodes, which represent crosslinking points, interconnected by polymer chains within a periodic simulation cell. For the random polymer network, nodes are positioned within a cubic unit cell using Poisson disk sampling, ensuring a minimum inter-node distance 1. In contrast, the uniform network features crosslinking points evenly distributed throughout the unit cell to maintain spatial uniformity. After node placement, the nodes are connected via polymer chains. Periodic boundary conditions (PBC) are applied to enforce continuous network replication across the simulation domain. The following sections detail the network construction methodology and key parameter definitions.

Chain Behavior of the Network Model

Under mechanical loading, hydrogels undergo macroscopic deformation, causing polymer chains to transform from initial random-coil configurations to highly extended and oriented conformations. During this deformation, entropic elasticity governs the mechanical response by restricting the statistical distribution of polymer chain conformations. As a result, the free energy of the mesoscopic network model is represented as the linear superposition of the free energies of individual polymer chains:

$$W = \sum_i W_i^{\mathrm{chain}} \tag{1}$$

To capture this behavior, the freely-jointed chain model incorporating Langevin statistics is utilized. This model describes the relationship between the free energy of individual polymer chains and their extension. The free energy of the i-th polymer chain, consisting of n_i Kuhn segments, is given by:

$$W_i^{\mathrm{chain}} = n_i kT \left(\frac{r_i}{R_i}\beta_i + ln\frac{\beta_i}{\sinh \beta_i}\right) \tag{2}$$

Here, $\beta_i = L^{-1}(r_i/R_i)$ is the inverse of the Langevin function $L(x) = \coth(x) - 1/x$, k is Boltzmann's constant, and T is the absolute temperature. The stretching force acting on the ends of the i-th chain is obtained by variational differentiation of the free energy of a polymer chain:

$$\boldsymbol{f}_i = \frac{\partial W_i^{\mathrm{chain}}}{\partial \boldsymbol{r}_i} = \frac{kT}{b_i}\beta_i \frac{\boldsymbol{r}_i}{\|\boldsymbol{r}_i\|} \tag{3}$$

Notably, as the end-to-end distance r_i approaches the chain contour length R_i (i.e., $r_i/R_i \to 1$), the theoretical internal stress diverges ($|\boldsymbol{f}_i| \to \infty$). Such divergence is physically inconsistent with the finite strength of real polymer chains. To address this issue, a damage factor η is introduced and defined as follows:

$$\eta = \begin{cases} 1, r_i/R_i < \lambda^c \\ 0, r_i/R_i \geq \lambda^c \end{cases} \tag{4}$$

Here, λ^c denotes the critical stretch at which polymer chains rupture. Beyond this critical stretch, the damage factor η reduces to zero, signifying polymer chain rupture. This damage criterion prevents unphysical stress divergence and allows for a realistic simulation of polymer chain rupture during deformation. **Figure S11**illustrates the fracture criterion for polymer chains under stretch. When the stretch of the i-th polymer chain r_i/R_i is below the critical threshold λ^c, the free energy increases monotonically with stretching. However, once r_i/R_i exceeds λ^c, the chain undergoes a dissipative transition, and the free energy abruptly drops to zero ($W^{\mathrm{chain}} \to 0$), corresponding to irreversible covalent bond rupture.

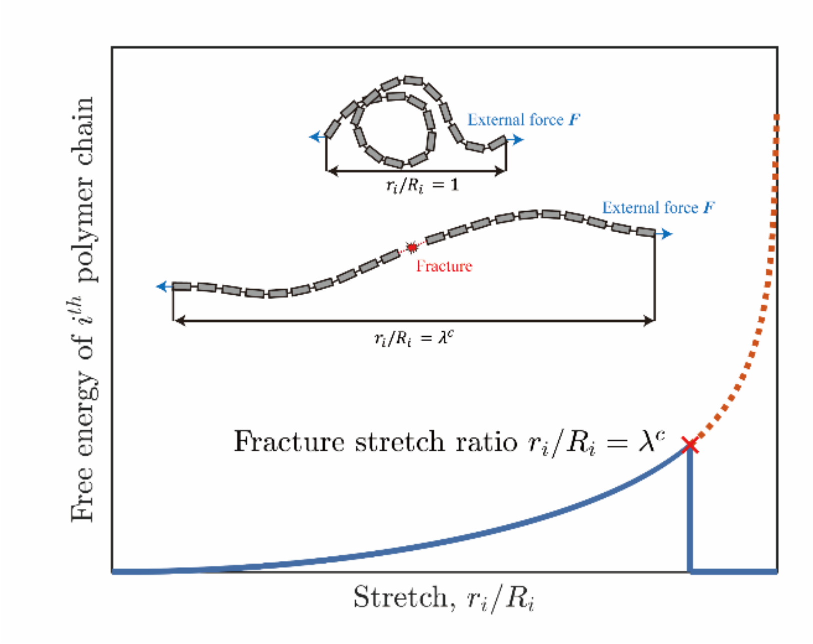

Figure S11. Illustration of the fracture criterion for polymer chains under stretching.

To enhance computational efficiency and simplify the evaluation of the inverse Langevin function, we implement the following high-accuracy analytical approximation:

$$L^{-1}(x) \approx \frac{x(3 - 1.00651x^2 - 0.962251x^4 + 1.47353x^6 - 0.48953x^8)}{(1-x)(1+1.01524x)} \quad (5)$$

This approximation exhibits a maximum error of only 0.076% within the interval $x \in [0,1]$, substantially reducing computational complexity compared to exact numerical methods while maintaining high accuracy.

Construction of the Network Model with Periodic Boundary Conditions (PBC)

Both uniform and random network models were constructed under periodic boundary conditions, with connectivity established using the Delaunay triangulation algorithm, as shown in **Figure S12.** In the random network topology, nodes were sampled using the Poisson disk sampling method to enforce volume exclusion between crosslinking points. **Figure S12a** illustrates the iterative exclusion process applied in a two-dimensional network configuration. Coordinate mapping was used to impose periodic boundary conditions and minimize the boundary-induced sparsity effect. When the minimum distance between a node and the boundary is less than the exclusion radius δ_D, its interaction extends across the periodic interface to corresponding periodic images, as illustrated by the pink disk in **Figure S12a**. This network generation approach ensures the continuity of the mesoscopic network topology across periodic interfaces. For the uniform network, nodes were uniformly distributed to maintain constant nearest-neighbor spacing. Then, the model domain as well as the mesh nodes were replicated into a 3×3×3 periodic array along the x-, y-, and z-directions and form a larger model domain (**Figure S12b**). Based on the replicated model, the Delaunay triangulation algorithm was subsequently used to generate a tetrahedral mesh for the expanded model. The geometric center of each tetrahedral element serves as a crosslinking point, which is connected to neighboring element centers via its four triangular faces. Each face forms a distinct linkage, creating a four-coordinated network structure. This configuration closely mirrors the tetrahedral crosslinking arrangement observed in an actual polyacrylamide (PAAm) hydrogel. Finally, only the crosslinking points

and polymer chains within the central domain of both uniform and random networks were preserved, resulting in a network model with periodic boundary conditions, as shown in **Figure S12C** and **S12D**.

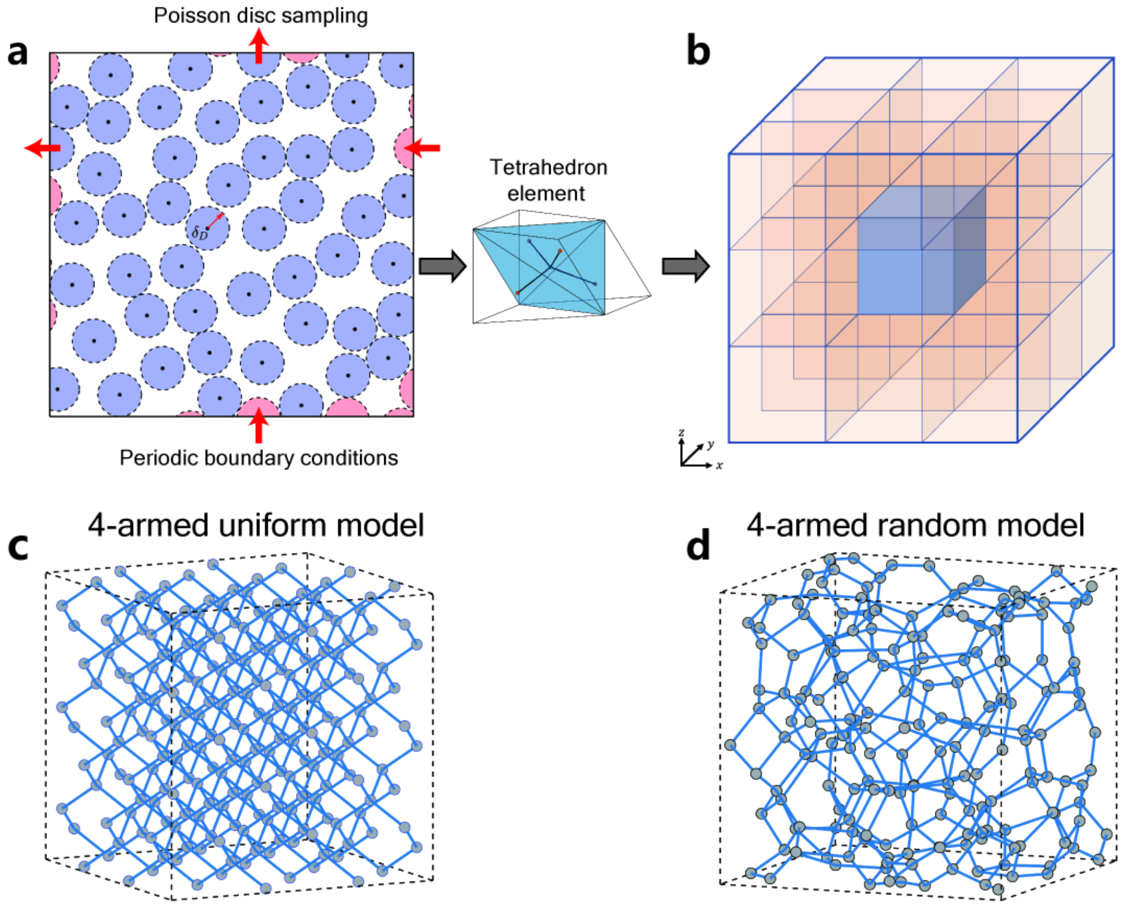

Figure S12. Mesoscopic network construction of hydrogels with uniform and random network structures.
(a) Schematic diagram of the network generation process illustrated in 2D. Crosslinking points are placed using Poisson disk sampling under periodic boundary conditions, with the minimum spacing of δ_D. A Delaunay tetrahedralization is then performed to construct a physically realistic, four-armed polymer network. **(b)** Schematic diagram of model assembly. **(c-d)** Uniform and random 4-armed network model.

Parameter Determination of the Network Model

In hydrogels prepared with AAm as monomer and MBAA as crosslinker, each crosslinker covalently bonds to four polymer chains, while each polymer chain connects two crosslinkers. Consequently, under the ideal condition where all functional groups fully react at X molar ratio of AAm to MBAA, each polymer chain contains $\bar{n}_m = X/2$ monomer units. The corresponding average contour length $\bar{R}$ of these polymer chains can then be expressed as:

$$\bar{R} = \bar{n}_m l_{\mathrm{AAm}} \tag{6}$$

where l_{AAm} represents the length of a single AAm monomer [2,3]. Based on mass conservation, the polymer chain density N can be derived as:

$$N = \frac{2\rho\phi_p}{2\bar{n}_m m_{\mathrm{AAm}} + m_{\mathrm{MBAA}}} \approx \frac{\rho\phi_p}{\bar{n}_m m_{\mathrm{AAm}}} \tag{7}$$

Here, ρ is the mass density of the PAAm hydrogel ($\rho = 1000\ \mathrm{kg/m^3}$), m_{AAm} is the mass of an AAm molecule ($m_{\mathrm{AAm}} = 1.185 \times 10^{-25}\ \mathrm{kg}$), and m_{MBAA} is mass of an MBAA molecule ($m_{\mathrm{MBAA}} = 2.570 \times 10^{-25}\ \mathrm{kg}$); ϕ_{p} represents the polymer mass fraction. Theoretically, for an ideal diamond-like lattice network, the average end-to-end distance $\bar{r}$ of chain segments is determined by the polymer chain density N as:

$$\bar{r} = \frac{\sqrt{3}}{4}\left(\frac{16}{N}\right)^{\frac{1}{3}} \tag{8}$$

Combining equations (6)-(8), an analytical expression for the Kuhn segment length b in the crosslinked network can be obtained:

$$b = \frac{\bar{r}^2}{\bar{R}} = \frac{3}{16\bar{n}_m l_{\mathrm{AAm}}}\left(\frac{16}{N}\right)^{\frac{2}{3}} \tag{9}$$

Table S2 summarizes the parameters and corresponding values used for the uniform and random network models; notably, all parameters except the polymer chain length distribution were kept constant across both models.

Table S2. The parameters used for uniform and random network model.

Parameters	ϕ_m(wt%)	M	n	R(nm)	$N(\times 10^{24}\mathrm{m}^{-3})$	$\bar{r}$	b
	10%	500:1	250	62.88	3.38	7.27	0.84

Numerical Implementation

The network model employs displacement-controlled loading, with the lower boundary ($\mathcal{B}_1$) fixed and axial displacement applied to the upper boundary ($\mathcal{B}_2$) to achieve uniaxial tension. The Newton-Raphson iterative method is adopted to numerically solve the model, with the core idea being the minimization of the total energy at each loading step, mathematically expressed by the residual vector ($\boldsymbol{R}$) and stiffness matrix ($\boldsymbol{K}$):

$$\boldsymbol{R} = \frac{\partial W}{\partial \boldsymbol{x}_{\mathrm{cross}}}, \boldsymbol{K} = \frac{\partial^2 W}{\partial \boldsymbol{x}_{\mathrm{cross}}^2} \tag{10}$$

Here, $\boldsymbol{x}_{\mathrm{cross}}$ represents the three-dimensional spatial coordinates of crosslinking nodes within the network model. In each iteration, corrections to the coordinates of the crosslinking nodes are determined by solving the following equations, aiming to reduce the total energy of the system:

$$\Delta\boldsymbol{x}_{\mathrm{cross}} = -\boldsymbol{K}^{-1} \cdot \boldsymbol{R} \tag{11}$$

When the maximum norm of the residual vector falls below the convergence criterion ($\|R\| < 10^{-6}$), the loading step is considered to have reached mechanical equilibrium. At each iteration, the stiffness matrix and residual vector are computed using Newton-Raphson iteration to minimize the potential energy of the system. Subsequently, the network is explicitly checked for any polymer chain ruptures. If polymer chain rupture is detected, the minimum energy of the system is recalculated based on the updated topology. The system advances to the next loading step only when both mechanical equilibrium and topological stability are simultaneously satisfied. The flowchart of numerical implementation is shown in **Figure S13**.

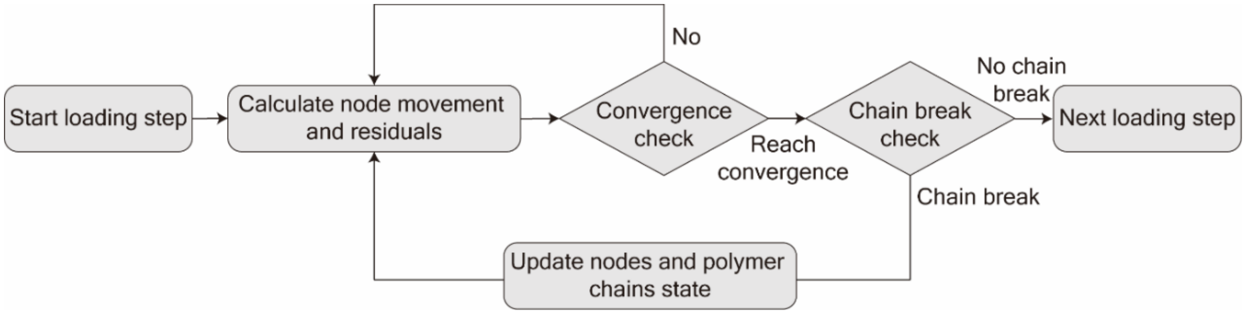

Figure S13. Flowchart of the numerical implementation of the network model.

The Cauchy stress along the tensile direction is calculated by summing the components of the force along the tensile direction of the polymer chain that passes through the periodic boundary during the tensile process. Its calculation is expressed as follows:

$$\sigma_s = \frac{1}{A_1}\sum_{j\in B_1} f_j = \frac{\lambda}{A_{10}}\sum_{j\in B_1} f_j \tag{12}$$

where A_1 and A_{10} is the current and initial sectional area of the network model along the stretching direction, respectively.

Validation of the Network Model

With the affine network deformation assumption, the free energy of the incompressible constitutive model can be expressed as:

$$W = NnkT\left(\sqrt{\frac{I_1}{3}}\frac{r_0}{R}\beta + ln\frac{\beta}{\sinh\beta}\right), \beta = L^{-1}\left(\sqrt{\frac{I_1}{3}}\frac{r_0}{R}\right) \tag{13}$$

where I_1 is the first invariant of the right Cauchy-Green deformation tensor, given by $I_1 = \lambda_1^2 + \lambda_2^2 + \lambda_3^2$, and the stretches along the three directions can be written as:

$$\lambda_1 = \lambda, \lambda_2 = \lambda_3 = \lambda^{-\frac{1}{2}} \tag{14}$$

The nominal stress-stretch relationship under uniaxial tension loading can therefore be written as:

$$P_{11} = \frac{NkTr_0}{\sqrt{3I_1 b}}L^{-1}\left(\sqrt{\frac{I_1}{3}}\frac{r_0}{R}\right)(\lambda - \lambda^{-2}) \tag{15}$$

As shown in **Figure S14**, the nominal stress–strain response of the uniform network, obtained through numerical simulation, demonstrates good agreement with the continuum-level theoretical prediction. This consistency validates the reliability of the mesoscale network modeling in capturing the constitutive behavior of polymer networks.

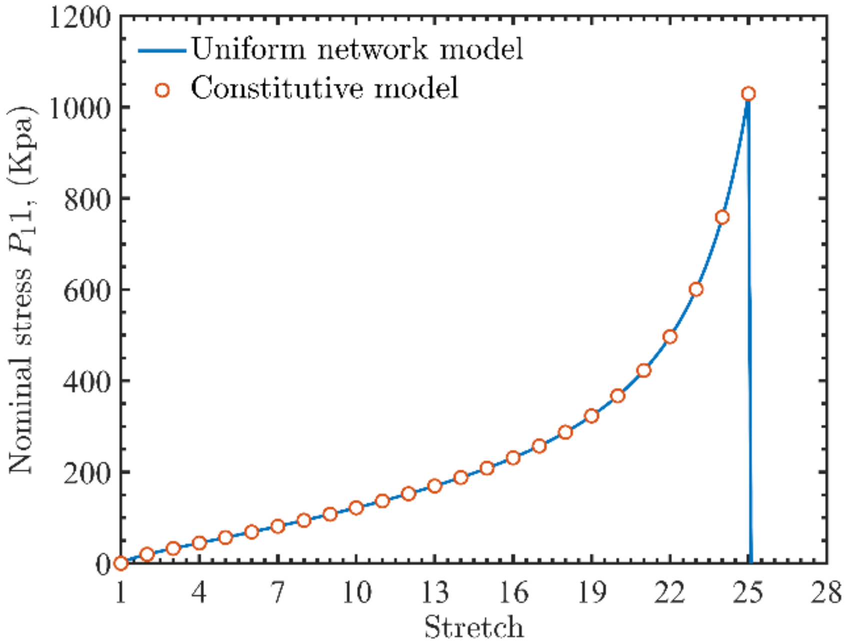

Figure S14. Comparison of simulation and theoretical results.
The uniform network model and the constitutive model exhibit identical stress responses under identical loading conditions.

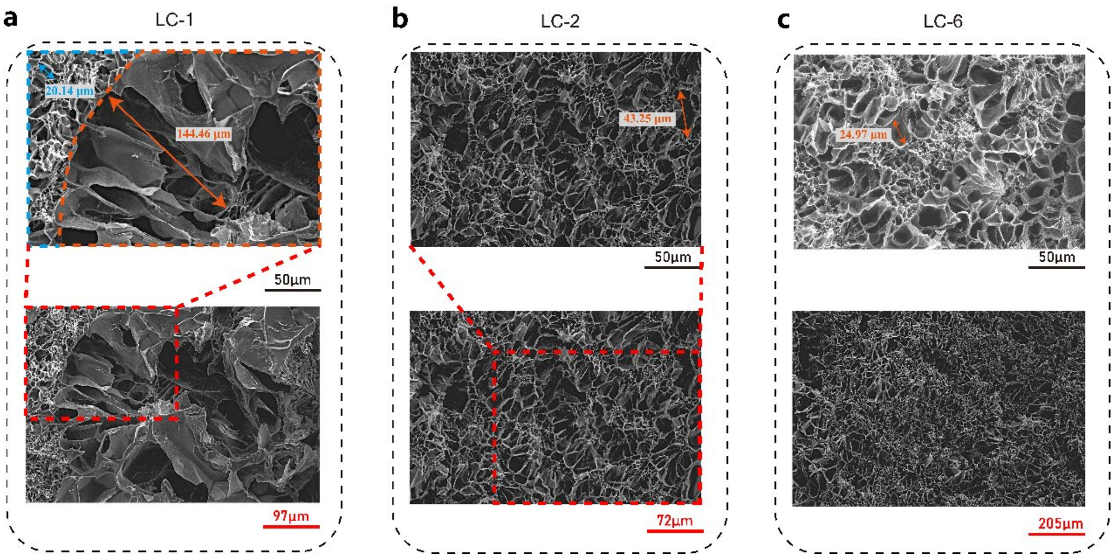

Figure S15. Low-magnification SEM images and corresponding enlarged views of hydrogels after different numbers of repeated swelling–curing cycles (LC-1, LC-2, and LC-6).

REFERNENCES

1. Araujo, L. M., Kryven, I., and Brassart, L. (2024). Micromechanical modelling of rubbery networks: The role of chain pre-stretch. International Journal of Non-Linear Mechanics 166, 104834 https://doi.org/10.1016/j.ijnonlinmec.2024.104834.

2. Lei, J., Xu, S., Li, Z., and Liu, Z. (2020). Study on Large Deformation Behavior of Polyacrylamide Hydrogel Using Dissipative Particle Dynamics. Front. Chem. 8, 115 https://doi.org/10.3389/fchem.2020.00115.

3. Lei, J., Li, Z., Xu, S., and Liu, Z. (2021). A mesoscopic network mechanics method to reproduce the large deformation and fracture process of cross-linked elastomers. Journal of the Mechanics and Physics of Solids 156, 104599 https://doi.org/10.1016/j.jmps.2021.104599.
